# Effect of the Expression of *ELOVL5* and *IGFBP6* Genes on the Metastatic Potential of Breast Cancer Cells

**DOI:** 10.3389/fgene.2021.662843

**Published:** 2021-06-02

**Authors:** Sergey Nikulin, Galina Zakharova, Andrey Poloznikov, Maria Raigorodskaya, Daniel Wicklein, Udo Schumacher, Stepan Nersisyan, Jonas Bergquist, Georgy Bakalkin, Lidiia Astakhova, Alexander Tonevitsky

**Affiliations:** ^1^Faculty of Biology and Biotechnologies, National Research University Higher School of Economics, Moscow, Russia; ^2^Scientific Research Centre Bioclinicum, Moscow, Russia; ^3^School of Biomedicine, Far Eastern Federal University, Vladivostok, Russia; ^4^Institute of Anatomy and Experimental Morphology, University Medical Center Hamburg-Eppendorf, Hamburg, Germany; ^5^Department of Chemistry – BMC, Uppsala University, Uppsala, Sweden; ^6^Department of Pharmaceutical Biosciences, Uppsala University, Uppsala, Sweden; ^7^School of Life Sciences, Immanuel Kant Baltic Federal University, Kaliningrad, Russia; ^8^Laboratory of Microfluidic Technologies for Biomedicine, Shemyakin-Ovchinnikov Institute of Bioorganic Chemistry RAS, Moscow, Russia

**Keywords:** breast cancer, *ELOVL5*, *IGFBP6*, matrix metalloproteinases, cell–cell contacts, MDA-MB-231

## Abstract

Breast cancer (BC) is the leading cause of death from malignant neoplasms among women worldwide, and metastatic BC presents the biggest problems for treatment. Previously, it was shown that lower expression of *ELOVL5* and *IGFBP6* genes is associated with a higher risk of the formation of distant metastases in BC. In this work, we studied the change in phenotypical traits, as well as in the transcriptomic and proteomic profiles of BC cells as a result of the stable knockdown of *ELOVL5* and *IGFBP6* genes. The knockdown of *ELOVL5* and *IGFBP6* genes was found to lead to a strong increase in the expression of the matrix metalloproteinase (MMP) *MMP1*. These results were in good agreement with the correlation analysis of gene expression in tumor samples from patients and were additionally confirmed by zymography. The knockdown of *ELOVL5* and *IGFBP6* genes was also discovered to change the expression of a group of genes involved in the formation of intercellular contacts. In particular, the expression of the *CDH11* gene was markedly reduced, which also complies with the correlation analysis. The spheroid formation assay showed that intercellular adhesion decreased as a result of the knockdown of the *ELOVL5* and *IGFBP6* genes. Thus, the obtained data indicate that malignant breast tumors with reduced expression of the *ELOVL5* and *IGFBP6* genes can metastasize with a higher probability due to a more efficient invasion of tumor cells.

## Introduction

Today, breast cancer (BC) is the most common malignant neoplasm in women worldwide (Bray et al., 2018). More than 2 million new cases of this disease are registered in the world every year. Despite the decrease in mortality from BC that has been outlined in recent years, it still remains the leading cause of death among women from cancer (Bray et al., 2018). More than 600,000 women die from BC in the world annually (Bray et al., 2018).

One of the main problems in the treatment of BC is relapse after primary treatment. According to recent statistics, relapse develops in about 40% of patients (Gerber et al., 2010; Lafourcade et al., 2018). Moreover, about one-third of the cases are local relapses, and two-thirds of the cases are distant metastases (Gerber et al., 2010; Lafourcade et al., 2018). Generally, treatment of patients with distant metastases is symptomatic and is not aimed at the complete cure of the disease (Gerber et al., 2010; Redig and McAllister, 2013).

In order to predict BC relapse earlier, methods for high-throughput analysis of gene expression revealed transcriptomic prognostic gene signatures (Hyams et al., 2017; Kwa et al., 2017). Today, the most popular commercially available transcriptomic test systems for BC used in clinical practice are Oncotype DX, Prosigna, and MammaPrint (Hyams et al., 2017; Kwa et al., 2017). On the one hand, utilization of transcriptomic test systems in clinical practice makes it possible to identify a group of patients with low risk of relapse and to avoid prescription of excessive treatment for them, which significantly improves the quality of life and reduces healthcare costs. On the other hand, their use allows early identification of patients with a high risk of distant metastases and justifies utilization of more intensive treatment protocols that reduce the risk of relapse. However, it should be noted that the need to create new, more advanced test systems is evidenced by the fact that the results of various tests available on the market do not agree well with each other when applied to the same group of patients (Bartlett et al., 2016).

Previously, our research group created its own classifier to identify patients with high risk of distant BC metastases, based on measuring the expression of only two genes (Galatenko et al., 2015). A fundamentally different approach to the selection of genes included in the consideration was used (Samatov et al., 2017; Galatenko et al., 2018). Traditionally, only genes with high individual information content were used in such gene signatures (those genes whose expression levels differ significantly between groups with favorable and unfavorable prognosis). At the same time, genes whose average expression did not differ significantly between groups with different prognosis were also used to construct this classifier. It was shown that taking the expression levels of such genes together with other genes into account can significantly improve the quality of classification. According to the obtained results, the most informative pair was the *ELOVL5*–*IGFBP6* gene pair (high expression of *ELOVL5* and *IGFBP6* corresponded to favorable prognosis). Previously, these genes had not been associated with the risk of BC metastases and, individually, do not have strong predictive power (i.e., it is not possible to assess the risk of relapse accurately based on the expression of just one of these genes). However, on the basis of the analysis of large microarray dataset of BC samples (kmplot.com), it can be concluded (Supplementary Figure 1) that high expression of each *ELOVL5* [hazard ratio (HR) = 0.54, *p* < 0.001] and *IGFBP6* (HR = 0.76, *p* < 0.001) messenger RNAs (mRNAs) is associated with better distant metastasis-free survival (DMFS) (Györffy et al., 2010).

Moreover, previously, *ELOVL5* and *IGFBP6* genes seemed to be unrelated to each other, and the reason for the observed synergism of the levels of expression of these two genes in the prediction of BC relapse was unclear. *ELOVL5* is one of the elongases of polyunsaturated fatty acids (PUFAs) located in the membrane of the endoplasmic reticulum (Leonard et al., 2000; Wang et al., 2008; Moon et al., 2009), and *IGFBP6* is a secreted protein that binds to insulin-like growth factors (IGFs) preventing their action on cells (Bach et al., 2013; Bach, 2015).

The spread of tumor cells throughout the body occurs during a multistep invasive-metastatic cascade, which consists of several stages (Samatov et al., 2015; Lambert et al., 2017). The aim of this work was to study the effect of the expression of the *ELOVL5* and *IGFBP6* genes on the features of BC cells associated with metastasis including the changes in the transcriptome and proteomic profiles as well as phenotypic traits.

## Materials and Methods

### Analysis of Transcriptomic Databases

The Cancer Cell Line Encyclopedia (CCLE) database was analyzed to select a BC cell line suitable for knockdown of the studied genes (Barretina et al., 2012).

The following datasets (Supplementary Table 1) from the Gene Expression Omnibus (GEO) were used for correlation analysis: GSE102484 (Cheng et al., 2017), GSE22220 (Camps et al., 2008), GSE3494 (Miller et al., 2005), GSE58644 (Miller et al., 2005), and GSE6532 (Loi et al., 2008). We also used data obtained by the METABRIC consortium (Cerami et al., 2012; Curtis et al., 2012) and The Cancer Genome Atlas (TCGA) program (Weinstein et al., 2013).

TAC 4.0 software (Thermo Fisher Scientific) was applied to preprocess raw data from Affymetrix microarrays. To carry out correlation analysis and statistical data processing, we employed the R 3.5 programming language with the RStudio 1.1 integrated development environment. The values of the Pearson correlation coefficient *R* and the *p*-values (the significance of the difference of *R* from zero) were calculated using the “cor.test” function. Correction for multiple comparisons was performed with the Benjamini–Hochberg method. The correlation coefficients with *p* < 0.05 were considered significant.

### Cell Culture

Human MDA-MB-231 BC cells were cultured in a complete cell culture medium consisting of Dulbecco’s modified Eagle’s medium (DMEM) high glucose (Gibco) supplemented with 10% vol. fetal bovine serum (Gibco), 2 mM L-glutamine (PanEco), and 1% vol. penicillin-streptomycin solution (Gibco). The cells were incubated in a cell culture incubator (37°C, 5% CO_2_) MCO-18AC (Sanyo). Subcultivation was performed every 2–3 days using trypsin–ethylenediaminetetraacetic acid (EDTA) solution (PanEco). Photomicrographs of the cells were obtained using an inverted Primo Vert microscope (Carl Zeiss). Cells were counted after trypan blue (Gibco) staining using Countess automated cell counter (Invitrogen) according to the manufacturer’s protocol.

To obtain three-dimensional spheroids, 96-well plates with low adhesion and a U-shaped bottom (Corning) were used. Two hundred microliters of cell suspension was added to each well of the plate. Then, the plate was incubated for 96 h in a cell culture incubator (37°C, 5% CO_2_) MCO-18AC (Sanyo). Photos of spheroids were obtained using an inverted microscope Axio Observer Z1 (Carl Zeiss). The experiment was performed independently three times. Each time a different number of cells per well was used (3,000, 5,000, and 6,000).

### Stable Knockdown of ELOVL5 and IGFBP6 Genes

Two cultures of MDA-MB-231 cells with reduced expression of messenger RNA (mRNA) of the *IGFBP6* gene (Supplementary Figure 2) were generated earlier (Nikulin et al., 2018). In this work, only MDA-MB-231 (IGFBP6_2) cells with the most pronounced decrease in *IGFBP6* gene expression were used as the cells with the *IGFBP6* gene knockdown. Stable knockdown of *ELOVL5* gene was performed similarly using RNA interference (Schwankhaus et al., 2014; Maltseva et al., 2020). DNA oligonucleotides selected for the target sequences in the *ELOVL5* gene were ligated into the pLVX short hairpin RNA 1 (shRNA1) lentiviral vector (Clontech Laboratories) according to the manufacturer’s protocol. We used two different target sequences with their own set of DNA oligonucleotides (Supplementary Table 2). To obtain the control MDA-MB-231 (LUC) cells, we used the same lentiviral vector pLVX shRNA1 containing shRNA to the *Photinus pyralis* firefly luciferase gene. Viral particles were obtained in the form of cell-free supernatants using transient transfection of HEK-293T cell line according to the previously described method (Weber et al., 2010, 2012). Supernatants were collected 24 h after transfection, filtered using 0.45-μm syringe filters, and stored at −80°C. Then, 5 × 10^4^ MDA-MB-231 cells were cultured in the wells of a 24-well culture plate in 0.5 ml of cell culture medium. After 24 h, 10 μl of the supernatant containing viral particles was added to the wells, and the plate was placed in a cell culture incubator for 24 h. Then, the cell culture medium was changed, and the cells were incubated for another 24 h. After that, the selection with 1 μg/ml puromycin (Gibco) was carried out for 2 weeks.

### Real-Time PCR

Real-time PCR was used to assess changes in the expression of individual genes as a result of the knockdown of the studied genes *ELOVL5* and *IGFBP6* (Nikulin et al., 2018). Cells of the studied lines were plated into six-well plates at 5 × 10^5^ cells per well in 2.5 ml of complete culture medium and incubated in a CO_2_ incubator (37°C, 5% CO_2_) for 48 h. Next, the cell culture medium was removed from the wells, and the cells were washed three times with cold (4°C) Dulbecco’s phosphate-buffered saline (DPBS) solution (PanEco). The cells were then lysed using QIAzol Lysis Reagent (QIAGEN). Seven hundred microliters (700 μl) of QIAzol Lysis Reagent solution (QIAGEN) was added to each well and incubated at room temperature for 5 min. Then, the contents of the wells were thoroughly mixed by pipetting and transferred into microtubes, which were stored at −80°C before RNA isolation.

RNA isolation was performed using miRNeasy Micro Kit (QIAGEN) according to the manufacturer’s protocol. RNA concentration was measured with a NanoDrop ND-1000 spectrophotometer (Thermo Fisher Scientific). The quality of the isolated RNA (no degradation) was assessed using Experion bioanalyzer (Bio-Rad). Only the samples with RNA integrity number (RIN) ≥ 7 were used.

Reverse transcription of RNA was performed using the MMLV RT kit (Evrogen) according to the manufacturer’s protocol. The obtained complementary DNA (cDNA) samples were stored at −20°C. qPCRmix-HS SYBR (Evrogen) was used for RT-PCR performed with DTprime detecting amplifier (DNA Technology).

The oligonucleotide primers used for RT-PCR were designed based on the mRNA sequences of the studied genes from the University of California Santa Cruz (UCSC) Genome Browser database (Kent et al., 2002). Primer selection was performed using Primer-BLAST software (Ye et al., 2012). The possibility of the formation of secondary structures (hairpins), homo- and heterodimers by the primers, was assessed using OligoAnalyzer 3.1 software (Owczarzy et al., 2008). *EEF1A1* and *HUWE1* were selected as reference genes (Maltseva et al., 2013). The sequences of the primers used, the lengths of the resulting amplicons, and the values of the amplification efficiencies are presented in Supplementary Table 3. The evaluation of the differences in the expression of the selected genes in the cells with knockdown of the *ELOVL5* and *IGFBP6* genes in comparison with the control MDA-MB-231 cells was carried out using the software REST 2009 v.2.0.13 (Pfaffl et al., 2002; Vandesompele et al., 2002). For each group, three independently obtained samples of RNA were used to assess expression levels of the selected genes.

### Western Blotting

Western blotting was used to evaluate the efficiency of the knockdown of the studied genes at protein level. To assess the knockdown of the *ELOVL5* protein, cells were lysed in radioimmunoprecipitation assay (RIPA) buffer; then, the protein concentration was measured using Pierce BCA Protein Assay kit (Thermo Fisher Scientific) according to the manufacturer’s instructions. Electrophoresis was performed in polyacrylamide gel (PAAG) (12%). Transfer to the polyvinylidene fluoride (PVDF) membrane was performed using Trans-Blot Turbo transfer system (Bio-Rad) according to the manufacturer’s instructions. The membrane was then blocked in 3% bovine serum albumin (BSA) solution in TBST (Tris-buffered saline, 0.1% Tween 20) for 1 h and incubated with rabbit primary antibodies to ELOVL5 protein (Abcam, ab205535) overnight at 4°C. Then, the membrane was washed in TBST solution and incubated with secondary goat antibodies to rabbit immunoglobulins conjugated with peroxidase. Clarity Western ECL Substrate (Bio-Rad) was used as a substrate for peroxidase. The resulting membrane was photographed using Gel Doc XR+ gel documenting station (Bio-Rad).

Since IGFBP6 is a secreted protein, serum-free medium samples after incubation with the cells for 24 h were analyzed to assess IGFBP6 protein knockdown. A similar Western blotting protocol was used (nonfat dry milk was used to block the membrane instead of BSA) with primary antibodies to the IGFBP6 protein (Abcam, ab109765). Samples were normalized to the number of cells.

Western blotting analysis for each protein was performed independently two times.

### Cell Proliferation Assay

The protocol of the used MTT cell proliferation assay has been published earlier (Nikulin et al., 2018). The proliferation rate was estimated as:

$$R_{72/24}=\frac{A_{72}-O}{A_{24}-O},$$

where *R*_*72/24*_ —the ratio of the number of cells in a well after 72 h to the number of cells after 24 h from seeding; *A*_24_,*A*_72_—the absorption value in the wells with the studied cells after 24 and 72 h; *O* - mean background absorption. The experiment was carried out in six replicas. Student’s *t*-test was used to determine the statistical significance of the observed differences.

### Apoptosis Assay

To study the activation of apoptosis, Dead Cell Apoptosis Kit with annexin V Alexa Fluor^TM^ 488 and propidium iodide (PI) (Thermo Fisher Scientific) were employed according to the manufacturer’s instructions. The cell suspension was centrifuged at 500 × *g* for 3 min, and the supernatant was collected. Then, the cells were resuspended in 100 μl of the buffer solution for annexin binding, and 5 μl of annexin V conjugate with Alexa Fluor 488 (AV) and 1 μl of propidium iodide (PI) solution with concentration of 100 μg/ml were added. After that, the cells were incubated at room temperature in a dark place for 15 min. Then, 400 μl of the buffer solution for annexin binding was added to the suspension, microtubes were transferred onto ice, and the samples were analyzed with a CytoFLEX flow cytometer (Beckman Coulter). The experiment was performed independently three times.

Analysis of raw data was carried out using FlowJo 10.6.1 software. As a result, the proportions of the entire cell population were obtained, corresponding to living cells (AV−PI−), cells at an early stage of apoptosis (AV+PI−), dead cells, including those at the late stages of apoptosis (AV+PI+), and nuclear fragments without cell membranes that can result from necrosis (AV−PI+) (Sawai and Domae, 2011). Further statistical data processing was carried out using R 3.5 programming language with RStudio 1.1 integrated development environment. Analysis of variance (ANOVA) was used to determine the statistical significance of the observed differences, followed by determination of *p*-values in pairwise comparisons using Tukey’s test. The differences were considered significant if the *p* < 0.05.

### Cell Migration Assay

Migration activity of the cells was measured by scratch assay. One hundred microliters of culture medium containing 3 × 10^4^ cells were added to each well of a 96-well plate. After that, the plate was incubated in a CO_2_ incubator (37°C, 5% CO_2_) overnight. Then, mitomycin C (Kyowa) was added to each well to the final concentration of 10 μg/ml for 2 h to stop proliferation. After that, scratches were made at the center of the wells using a 200-μl pipette tip, and cell culture medium was changed. Then, the plates were placed into a cell culture incubator. Each well was microphotographed at different time points (0, 4, 8, and 10 h) using a SpectraMax i3 plate reader (Molecular Devices). The experiment was carried out in 20 replicas.

ImageJ software was used to calculate the area of the scratches. Then, the dependence of the scratch area on time was plotted for each well, and the migration rate was estimated as the slope coefficient of the resulting straight line. The wells where coefficient of determination *R*^2^ of the fitted straight line was < 0.95 were removed from further analysis. To determine the statistical significance of the observed differences, Mann–Whitney *U* test was applied.

### Transcriptomic Analysis

Transcriptomic analysis of the generated cell cultures was performed using Human Transcriptome Array 2.0 microarrays (Affymetrix) according to the manufacturer’s procedure. For each group, three independently obtained samples of RNA were used.

Raw data were processed using TAC 4.0 software (Thermo Fisher Scientific) using the RMA algorithm. To assess the statistical significance of differences in gene expression, ANOVA FDR *p*-values with threshold level of 0.05 were used. Further data processing was conducted using R 3.5 programming language with the RStudio 1.1 integrated development environment.

Statistical significance of the intersection between regulated genes (the probability that the intersection is a random event) after the knockdown of *ELOVL5* and *IGFBP6* genes was determined by permutation test (Nikitin et al., 2019; Sorokin et al., 2020). To determine the distribution of the number of the genes that significantly change their expression in the same direction after the knockdown of *ELOVL5* and *IGFBP6* genes in case of completely independent changes, gene names were randomly permuted 1,000,000 times. The fold changes and *p*-values were conserved, and the size of the overlap between genes significantly regulated in the same direction for each generated random gene set was measured.

Analysis of the enriched biological processes among the genes with increased and decreased expression was carried out using gene ontology (GO) database (Ashburner et al., 2000; Gene and Consortium, 2019) and “topGO” package for R programming language. The results were obtained using “weight01” algorithm; *p*-values were calculated using Fisher’s exact test.

Pathway activation levels (PALs) were calculated with Oncobox Library (Sorokin et al., 2021) with the default set of pathway databases. Comparison of PALs between *ELOVL5*/*IGBFP6* knockdown cells with control ones was done with Student’s *t*-test; Benjamini–Hochberg procedure was used to adjust *p*-values.

### Proteomic Analysis

For proteomic analysis, cells were lysed with 3% sodium deoxycholate (SDC) solution in bicarbonate buffer (50 mM ammonium bicarbonate in water). The lysates were incubated for 15 min at 80°C, followed by sonication. Then, the disulfide bonds in the proteins were reduced with dithiothreitol (DTT) and alkylated with iodoacetamide (IAA). The resulting protein mixture was digested with Trypsin Gold (Promega) at 37°C overnight. Then, SDC was removed from the mixture by precipitation with trifluoroacetic acid. The resulting mixture of peptides was purified using ZipTips (Merck Millipore) according to the manufacturer’s protocol. Then, the samples were dried and dissolved in 0.1% vol. formic acid solution. The resulting peptides were analyzed using a nano-high performance liquid chromatography tandem mass spectrometry (nano-HPLC-MS/MS) system coupled with a Q Exactive Orbitrap mass spectrometer (Thermo Fisher Scientific). Separation was carried out with a reversed-phase C_18_ column in gradient elution mode; the duration of the gradient was 150 min. Fragment spectra were obtained using collision-induced dissociation. For each group, three independently obtained samples of proteins were used.

Raw data were analyzed using MaxQuant 1.6 software (Tyanova et al., 2016a). The iBAQ algorithm was used to quantify the protein content (Schwanhäusser et al., 2011). Further data processing was carried out using Perseus 1.6 software (Tyanova et al., 2016b) and R 3.5 programming language with the RStudio 1.1 integrated development environment. To determine the statistical significance of the observed differences, Student’s *t*-test was used.

Enrichment analysis of biological processes and pathway analysis were performed as described above for transcriptomic analysis.

### Zymography

The utilized method for assessment of the activity of matrix metalloproteinases (MMPs) was described earlier (Toth and Fridman, 2001). Serum-free cell culture medium was sampled after 24 h of incubation with the cells. Then, electrophoresis was performed in polyacrylamide gel containing 0.1% of gelatin. The resulting gel was incubated at 37°C overnight. Then, the gel was stained with Coomassie blue G-250 colloidal solution (Thermo Fisher Scientific). Clear zones in the stained gel correspond to the positions of active MMPs. The gel was photographed with Gel Doc XR+ gel documenting station (Bio-Rad). Zymography was performed independently two times.

## Results

### Stable Knockdown of ELOVL5 and IGFBP6 Genes

**FIGURE 1:**
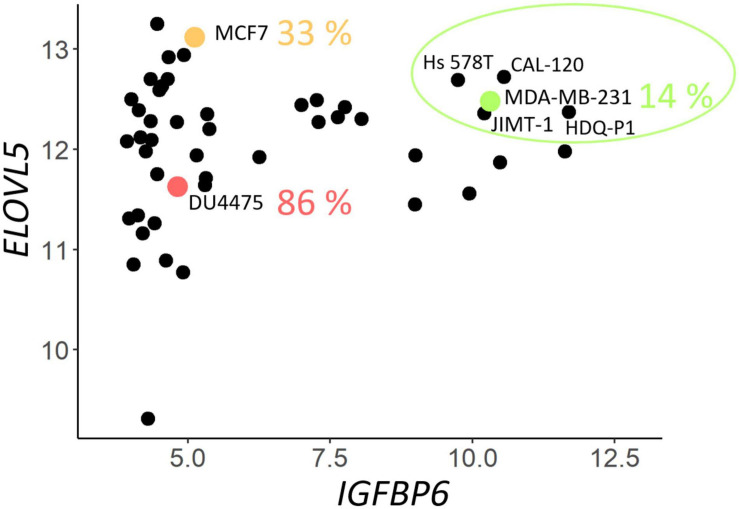
Expression (on the Affymetrix logarithmic scale) of the ELOVL5 and IGFBP6 genes in breast cancer cell lines [according to Cancer Cell Line Encyclopedia (CCLE) data (Barretina et al., 2012)]. The proportion of cases when some of the cell lines form metastases to lungs *in vivo* (immunodeficient mice) is indicated according to Valentiner et al. (2005).

To select suitable cell lines for the knockdown, a two-dimensional plot of the expression of *ELOVL5* and *IGFBP6* genes in BC cell lines according to publicly available database CCLE was constructed. It can be seen from the plot (Figure 1) that only a few cell lines have a sufficiently high expression of both studied genes (circled in green), and they are suitable ones for knockdown. Expression of major molecular markers in this group of cell lines is presented in Supplementary Figure 3 (Dai et al., 2017). All these cells are estrogen and progesterone receptor negative, and only one of them is HER2 positive. Among these candidates for the knockdown, there was only one cell line that is often used as a model of triple negative BC. This cell line is MDA-MB-231, and it was chosen as the original cell model in this work.

**FIGURE 2:**
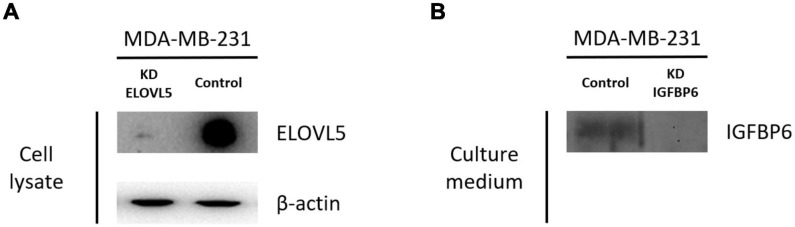
Results of analysis of the expression of ELOVL5 and IGFBP6 proteins by Western blotting. **(A)** Relative expression of the ELOVL5 protein in the MDA-MB-231 (ELOVL5) cell line compared to the control cell line. **(B)** Relative content of the IGFBP6 protein in the conditioned cell culture medium of MDA-MB-231 (IGFBP6) cell line compared to the control cell line (samples were normalized to the number of cells).

Two cultures of MDA-MB-231 cells with a stable knockdown of the *ELOVL5* gene were generated in this work (Table 1 and Supplementary Figure 2). For further analysis, only MDA-MB-231 (ELOVL5_2) cells with the most pronounced decrease in *ELOVL5* gene expression were used as the cell line with the *ELOVL5* gene knockdown. Decreased expression of ELOVL5 and IGFBP6 proteins in MDA-MB-231 cell lines with stable knockdown of these genes was additionally qualitatively confirmed by Western blotting (Figure 2 and Supplementary Figure 4).

**TABLE 1:** Relative expression of the *ELOVL5* gene in the cells with a stable knockdown (shRNA) of the *ELOVL5* gene compared to the control cells MDA-MB-231 (LUC).

Cell line	Relative expression	95 % confidence interval	*p*-value
MDA-MB-231 (ELOVL5_1)	*ELOVL5:* 0.532	0.382–0.753	0.026
MDA-MB-231 (ELOVL5_2)	*ELOVL5:* 0.244	0.160–0.369	0.031

### Cell Proliferation

**FIGURE 3:**
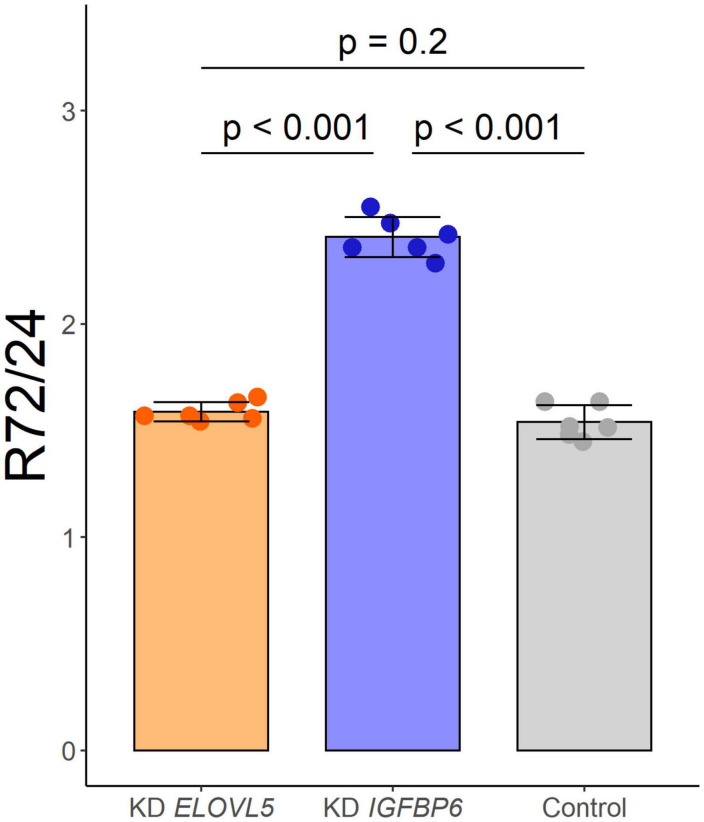
Effect of the knockdown of *ELOVL5* and *IGFBP6* on the proliferation rate of MDA-MB-231 cells (data are mean ± SD, *n* = 6 wells per group). *R*_72_/24− the ratio of the number of cells in a well after 72 h to the number of cells after 24 h from seeding.

As a result of the analysis of the effect of knockdown of the *ELOVL5* and *IGFBP6* genes on the proliferation rate of MDA-MB-231 cells, it was shown (Figure 3) that the knockdown of the *IGFBP6* gene leads to a significant increase in the proliferation rate, while the knockdown of the *ELOVL5* gene did not statistically significantly change the proliferation rate.

### Apoptosis

**FIGURE 4:**
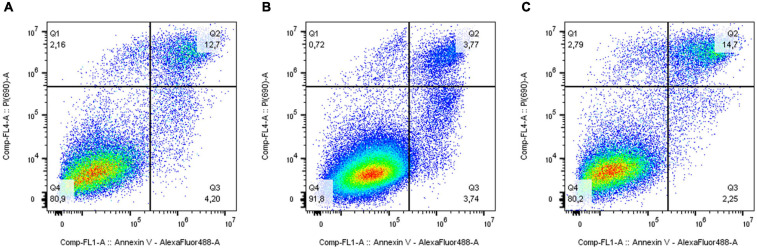
Effect of *ELOVL5* and *IGFBP6* knockdown on the activation of apoptosis. Two-dimensional plots of the integral fluorescence intensity of the annexin V conjugate with Alexa Fluor 488 dye (horizontal axis) and the integral fluorescence intensity of propidium iodide (vertical axis) in MDA-MB-231 cells with **(A)** *ELOVL5* and **(B)** *IGFBP6* genes knockdown, as well as in **(C)** controls cells.

The analysis of the activation of apoptosis (Figure 4 and Supplementary Figure 5) in MDA-MB-231 cells after knockdown of the *ELOVL5* gene showed that the number of dead cells, including the cells in the late stages of apoptosis, (AV+PI+) did not change in comparison to control cells (*p* = 0.66). At the same time, the knockdown of the *IGFBP6* gene led to a significant decrease in the proportion of dead (AV+PI+) cells in the population (by about three times, *p* = 0.006). In addition, the proportion of viable cells in the population increased significantly (by about 11%, *p* = 0.009). Interestingly, the knockdown of the *IGFBP6* gene led to a decrease in the proportion of nuclear fragments without cell membrane (AV−PI+), which can be formed as a result of necrosis (from 2.9 to 0.6%, *p* = 0.02). No significant changes in the proportion of cells at an early stage of apoptosis as a result of the knockdown of the *ELOVL5* and *IGFBP6* genes were found (ANOVA, *p* = 0.24).

### Cell Migration

**FIGURE 5:**
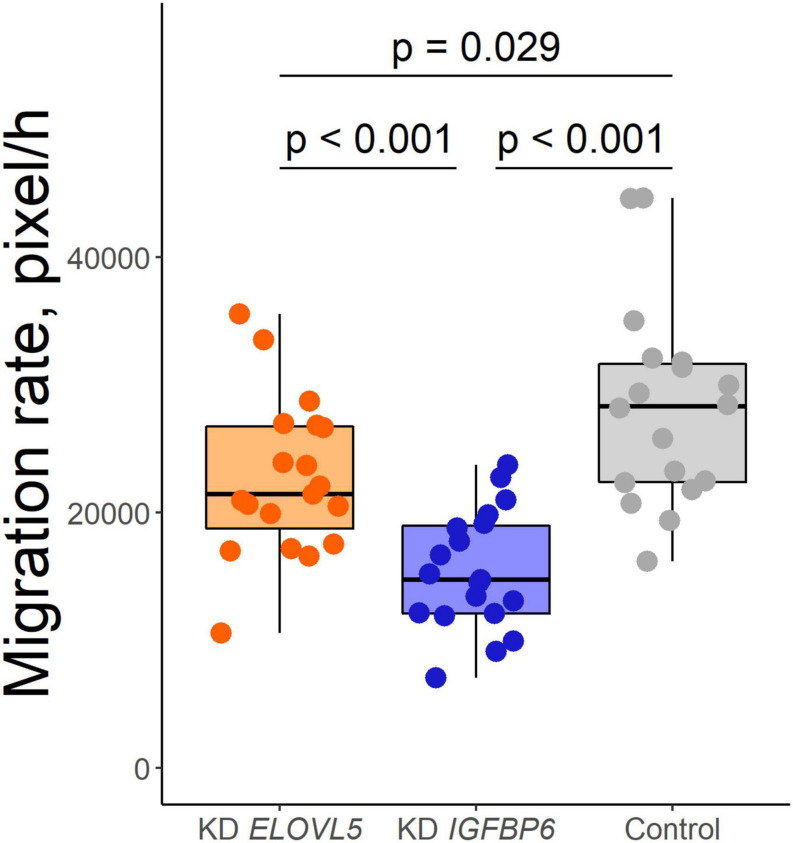
Boxplot of cell migration activity according to the scratch wound healing assay (*n* = 19, *n* = 19, and *n* = 18 wells for KD *ELOVL5*, KD *IGFBP6*, and control cells, respectively).

As a result of the analysis of the effect of knockdown of the *ELOVL5* and *IGFBP6* genes on the migration activity of MDA-MB-231 cells by scratch assay, it was shown (Figure 5) that the knockdown of the *IGFBP6* gene leads to a significant decrease in migration activity (by about 27%, *p* < 0.001), while the knockdown of the *ELOVL5* gene leads to a similar, but less pronounced effect (migration activity decreases by about 15%, *p* = 0.029).

### Aggregation Into 3D Cell Spheroids

**FIGURE 6:**
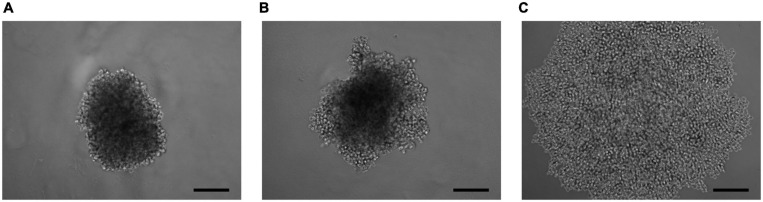
Photo of 3D cell spheroids (5,000 cells per well at zero time point) after 96 h from seeding consisting of **(A)** control cells MDA-MB-231 (LUC) and the cells with a stable knockdown of **(B)** *ELOVL5* and **(C)** *IGFBP6* genes. The scale bar length is 200 μm.

As a result of the analysis of the effect of knockdown of the *ELOVL5* and *IGFBP6* genes on the ability of MDA-MB-231 cells to form 3D spheroids, it was shown (Figure 6 and Supplementary Figure 6) that the knockdown of the *IGFBP6* gene leads to the inability of cells to form 3D spheroids. The knockdown of the *ELOVL5* gene resulted in MDA-MB-231 cells forming less dense 3D spheroids with rough edges.

### Transcriptomic Analysis

**FIGURE 7:**
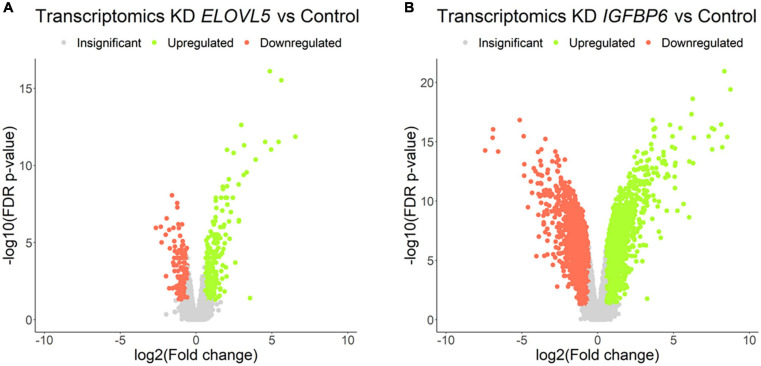
Volcano plots for comparative transcriptome analysis of the cells with a stable knockdown of **(A)** *ELOVL5* and **(B)** *IGFBP6* genes. Thresholds: Fc = 1.5, FDR *p*-value = 0.05.

Our own transcriptomic analysis of the generated cell cultures demonstrated good correlation between replicates (Supplementary Figure 7) and showed (Figure 7 and Supplementary Table 4) that the knockdown of the *ELOVL5* gene leads to a significant change in the expression of <2% of known genes, while the knockdown of the *IGFBP6* gene leads to a change in the expression of more than 16% of genes.

Among the genes with the most pronounced changes in mRNA expression, both in *ELOVL5* knockdown and *IGFBP6* knockdown cells, the *MMP1* and *MMP3* metalloproteinase mRNAs were found. After the knockdown of the *ELOVL5* gene, the content of mRNA of the *MMP1* and *MMP3* genes increased by 94 (FDR *p* = 1.4 × 10^–12^) and 7 (FDR *p* = 4.3 × 10^–7^) times, respectively, and after the knockdown of the *IGFBP6* gene by 244 (FDR *p* = 4.3 × 10^–14^) and 374 (FDR *p* = 4.0 × 10^–16^) times, respectively.

A significant change in the expression of the genes of MMPs *MMP1* and *MMP3* at mRNA level was additionally confirmed by RT-PCR. RT-PCR showed that the expression of the *MMP1* gene increased after the knockdown of the *ELOVL5* gene by about 76 times (*p* < 0.001) and after the knockdown of the *IGFBP6* gene by about 760 times (*p* = 0.028). It was not possible to quantify the ratio of *MMP3* gene expression levels in control cells and the cells with the knockdown of *ELOVL5* and *IGFBP6* genes using RT-PCR due to too low content of *MMP3* gene mRNA in control cells (the fluorescence intensity was below the threshold value after 40 amplification cycles). However, the registration of the PCR product was possible for cell lines with the knockdown of the *ELOVL5* and *IGFBP6* genes. Moreover, the threshold cycle value (Ct) for the cells with the knockdown of the *IGFBP6* gene (31.9; standard deviation, 0.1) was significantly less than for the cells with the knockdown of the *ELOVL5* gene (37.9; standard deviation, 1.0). Thus, it can be seen from the obtained data that the results of analysis of the levels of expression of the *MMP1* and *MMP3* mRNA using real-time PCR are in good agreement with the results of transcriptomic analysis by Affymetrix chips.

Further analysis identified a group of 364 genes with statistically significant change in mRNA expression in the same direction, both in the *ELOVL5* gene knockdown and in the *IGFBP6* gene knockdown cells. It has been shown (Supplementary Figure 8) that the size of this overlap is too high to be a random event (*p* < 0.001). As a result of the analysis of biological processes enriched for the genes from this group, it was shown that among the genes with reduced expression, there is a significant number of genes involved in the formation of adherens junctions (Table 2). On the other hand, among the genes with increased expression, there are several genes involved in the formation of other types of intercellular contacts, as well as in the regulation of the formation of cell–cell contacts.

**TABLE 2:** Selected enriched biological processes for the genes with a concordantly changed expression after knockdown of the *ELOVL5* and *IGFBP6* genes.

The genes with decreased expression		The genes with increased expression	
GO ID	Biological processes	GO ID	Biological processes
GO:0045216	Cell–cell junction organization	GO:0007156	Homophilic cell adhesion via plasma membrane adhesion molecules
GO:0034332	Adherens junction organization	GO:0016339	Calcium-dependent cell–cell adhesion via plasma membrane cell adhesion molecules
GO:0007157	Heterophilic cell–cell adhesion via plasma membrane cell adhesion molecules	GO:0007416	Synapse assembly
GO:0031102	Neuron projection regeneration	GO:2000651	Positive regulation of sodium ion transmembrane transporter activity
GO:0007155	Cell adhesion	GO:0045653	Negative regulation of megakaryocyte differentiation
GO:0003179	Heart valve morphogenesis	GO:1904837	Beta-catenin-TCF complex assembly
GO:0044331	Cell–cell adhesion mediated by cadherin	GO:0014829	Vascular smooth muscle contraction
GO:0060045	Positive regulation of cardiac muscle cell proliferation	GO:0032656	Regulation of interleukin-13 production
GO:0002921	Negative regulation of humoral immune response	GO:0022407	Regulation of cell–cell adhesion
GO:1903975	Regulation of glial cell migration	GO:0006335	DNA replication-dependent nucleosome assembly

Among the most pronounced changes in the levels of expression of cell adhesion molecules, one can distinguish a strong decrease in the expression of the *CDH11* gene as a result of the knockdown of the *IGFBP6* gene (approximately 119 times, FDR *p* = 9.1 × 10^–17^). A similar, but significantly smaller change in the expression of the *CDH11* gene was also found after the knockdown of the *ELOVL5* gene (approximately 3.4 times, FDR *p* = 1.5 × 10^–6^). In addition, as a result of the knockdown of the *ELOVL5* and *IGFBP6* genes, the expression of the *CLDN1* (*ELOVL5*: 3.4 times, FDR *p* = 9.2 × 10^–3^; *IGFBP6*: 2.0 times, FDR *p* = 4.2 × 10^–2^) and *DSP* (*ELOVL5*: 1.7 times, FDR *p* = 3.1 × 10^–3^; *IGFBP6*: 4.9 times, FDR *p* = 3.4 × 10^–11^) genes consistently decreased.

### Correlation Analysis

The analysis of the publicly available databases of transcriptomes of BC samples showed (Supplementary Table 5) that *MMP1* gene expression negatively correlates with *ELOVL5* gene expression (i.e., increases with a decrease in *ELOVL5* gene expression) in tumor samples from patients with ER+ BC in seven analyzed data sets (in total, 10 datasets of ER+ BC were analyzed) and in only one dataset of ER−BC patients (in total seven datasets of ER−BC were analyzed). In addition, *MMP1* gene expression negatively correlated with *IGFBP6* gene expression in the samples from four datasets of ER+BC and three datasets of ER−BC.

A weak negative correlation of the expression of the *MMP3* gene with the expression of the *ELOVL5* gene was observed only in one dataset of ER+BC. On the other hand, the correlation of the level of *MMP3* gene expression with the level of *IGFBP6* gene expression was positive in six datasets of ER+ tumors and in one dataset of ER− tumors.

According to the correlation analysis, the level of expression of the *CDH11* gene in the tumor tissue of patients with BC often positively correlates with the levels of expression of the *ELOVL5* and *IGFBP6* genes (*ELOVL5*: in four data sets of ER+ BC and in four data sets for ER− BC; *IGFBP6:* in seven data sets of ER+BC and three data sets of ER−BC). The only exception was the statistically significant weak negative correlation with the level of *IGFBP6* gene expression in one dataset of ER+ patients.

### Proteomic Analysis

**FIGURE 8:**
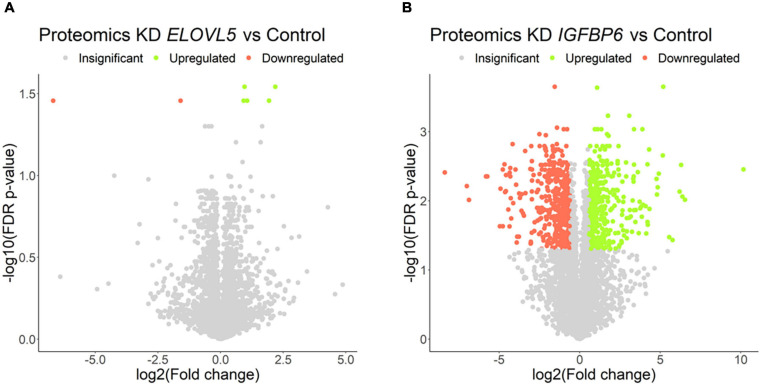
Volcano plots for comparative proteome analysis of the cells with a stable knockdown of **(A)** *ELOVL5* and **(B)** *IGFBP6* genes. Thresholds: Fc = 1.5, FDR *p*-value = 0.05.

**FIGURE 9:**
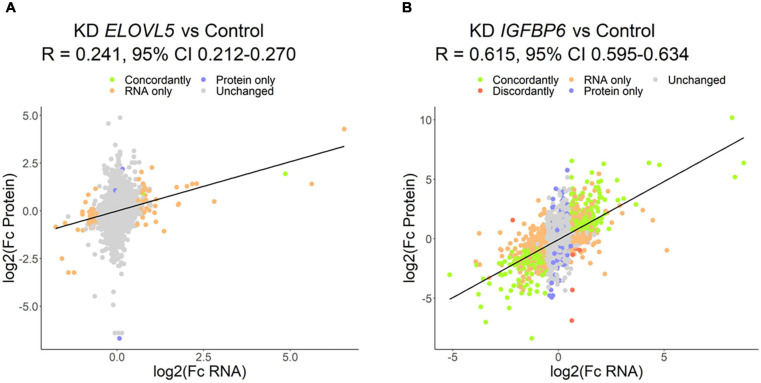
Correlation of the results of proteomic and transcriptomic analysis of the cells with a stable knockdown of the **(A)** *ELOVL5* and **(B)** *IGFBP6* genes.

The proteomic analysis demonstrated good correlation between replicates (Supplementary Figure 9). As a result, it was shown (Figure 8 and Supplementary Table 6) that the knockdown of the *ELOVL5* gene leads to an insignificant change in the expression of proteins in the cell (<0.2% of the total number of all measured proteins), while the knockdown of the *IGFBP6* gene leads to a significant change in expression of more than 20% of the measured proteins. The correlation analysis of the results (Figure 9) obtained during the study of transcriptomic and proteomic profiles revealed that in the case of the knockdown of the *IGFBP6* gene, there is a fairly high correlation of the results. In the case of the knockdown of the *ELOVL5* gene, despite the statistical significance, the correlation was low. This phenomenon can be explained by the lower sensitivity of proteomic analysis to small changes in expression as compared to a transcriptomic one.

The proteomic analysis confirmed a significant increase in the expression of the MMP1 protein after the knockdown of the *IGFBP6* gene (1,157 times, FDR *p* = 3.5 × 10^–3^) and a decrease in the DSP protein content (4.5-fold, FDR *p* = 1.6 × 10^–3^). At the same time, no decrease in the content of the OCLN protein was found (FDR *p* = 0.33). Moreover, the analysis of biological processes enriched for the proteins with altered expression after *IGFBP6* gene knockdown showed that among the genes with reduced expression, there is a significant group of genes involved in cell migration and adhesion (Table 3 and Supplementary Table 7). On the other hand, among the genes with increased expression, there are a lot of genes involved in cellular respiration and ribosome assembly.

**TABLE 3:** Selected enriched biological processes for the proteins with a significantly changed expression after knockdown of the *IGFBP6* gene.

The proteins with decreased expression		The proteins with increased expression	
GO ID	Biological processes	GO ID	Biological processes
GO:0044319	Wound healing, spreading of cells	GO:0000462	Maturation of SSU-rRNA from tricistronic rRNA transcript (SSU-rRNA, 5.8S rRNA, LSU-rRNA)
GO:0022617	Extracellular matrix disassembly	GO:0031167	rRNA methylation
GO:1903779	Regulation of cardiac conduction	GO:0009303	rRNA transcription
GO:0010812	Negative regulation of cell–substrate adhesion	GO:0070125	mitochondrial translational elongation
GO:0010771	Negative regulation of cell morphogenesis involved in differentiation	GO:0045333	Cellular respiration
GO:0001666	Response to hypoxia	GO:0010501	RNA secondary structure unwinding
GO:0030239	Myofibril assembly	GO:0045943	Positive regulation of transcription by RNA polymerase I
GO:0051155	Positive regulation of striated muscle cell differentiation	GO:0030490	Maturation of SSU-rRNA
GO:0007186	G-protein-coupled receptor signaling pathway	GO:0070126	Mitochondrial translational termination
GO:1900024	Regulation of substrate adhesion-dependent cell spreading	GO:0007005	Mitochondrion organization

### Pathway Activation Analysis

Pathway activation levels were calculated for both experimental conditions (*ELOVL5* knockdown vs control, *IGFBP6* knockdown vs control) and both transcriptomics and proteomics data (Supplementary Table 8).

The most activated pathway after the knockdown of *ELOVL5* gene according to the microarray analysis was “Reactome basigin interactions main pathway” (FDR *p* = 0.039). Basigin (CD147) is a cell surface protein that can activate the production of MMPs by adjacent cells (Nabeshima et al., 2006). In addition, basigin is known to promote progression of various cancers (Kanekura and Chen, 2010). On the other hand, the only significantly downregulated pathway in the cells with reduced expression of *ELOVL5* was “Reactome nectin/necl trans heterodimerization main pathway” (FDR *p* = 0.038). Nectins are well known cell adhesion molecules, and downregulation of this pathway is consistent with reduced cell adhesion after the knockdown of *ELOVL5* gene (Sakisaka et al., 2007). According to the proteomics analysis, there were no downregulated pathways, while the most upregulated one was the “Biocarta Erk and PI-3 kinase are necessary for collagen binding in corneal epithelia pathway (actin filament stabilization)” (FDR *p* = 0.048), indicating importance of these signals in the progression of BC (Chu et al., 2000; Ebi et al., 2013).

Based on the transcriptomics data, the most activated pathway after the knockdown of *IGFBP6* gene was “NCI Class IB PI3K non-lipid kinase events pathway (cAMP biosynthetic process)” (FDR *p* = 0.004). It is well known that PI3K signaling is often deregulated in cancer. Specifically, class IB PI3K is important for the proliferation of pancreatic cancer cells (Edling et al., 2010). Our results suggest that class IB PI3K can be important for the proliferation of BC cells, too. The most activated pathway after the knockdown of *IGFBP6* according to proteomic analysis was the “NCI validated transcriptional targets of AP1 family members Fra1 and Fra2 main pathway” (FDR *p* = 0.028). Fra-1 and Fra-2 are well-studied transcription factors important for the progression of BC. For example, Fra-1 can directly increase the expression of *MMP1* (Belguise et al., 2005), and Fra-2 promotes the invasion of BC cells (Schröder et al., 2010). On the other hand, the most downregulated pathways in the cells with reduced expression of *IGFBP6* according to transcriptomic analysis were integrin-linked kinase pathways (“ILK signaling pathway opsonization,” FDR *p* = 0.003; “ILK signaling pathway cell adhesion,” FDR *p* = 0.004; “ILK signaling pathway regulation of junction assembly at desmosomes,” FDR *p* = 0.004; “ILK signaling pathway wound healing,” FDR *p* = 0.004), which regulate cell adhesion, motility, and opsonization (Zheng et al., 2019). Downregulation of these pathways is consistent with observed reduced adhesion and motility of BC cells with the knockdown of *IGFBP6* gene. In addition, pathway analysis of proteomic data revealed inhibition of the “hypusine biosynthesis” pathway (FDR *p* = 0.035). Hypusine is a noncanonical amino acid containing only in two proteins: eIF5A1 and eIF5A2 (Muramatsu et al., 2016). Its modification leads to activation of the RhoA signaling pathway and increased cell motility (Muramatsu et al., 2016). Decreased cell migratory activity is consistent with the inhibition of this pathway.

Consistently with differential expression and GO terms enrichment analyses, the knockdown of *IGFBP6* led to a significantly higher number of altered pathways compared to *ELOVL5* case. Namely, 929 and 791 pathways were regulated upon *IGFBP6* knockdown for transcriptomics and proteomics data, respectively (adjusted *p* < 0.05), while only 5 and 3 pathways were identified upon *ELOVL5* knockdown. From these, three Reactome pathways were common for *ELOVL5* and *IGFBP6* knockdowns: “Reactome activation of MMPs main pathway” (upregulated upon both knockdowns, FDR *p* = 0.037 and FDR *p* = 0.003, respectively), “Reactome Basigin interactions main pathway” (upregulated upon both knockdowns, FDR *p* = 0.039 and FDR *p* = 0.019, respectively) and “Reactome Nectin/Necl trans heterodimerization main pathway” (downregulated upon both knockdowns, FDR *p* = 0.038 and FDR *p* = 0.003, respectively). While the analysis showed consistent results between transcriptomics and proteomics data upon *IGFBP6* knockdown (337 common activated pathways, *p* = 1.4 × 10^–10^), three pathways associated with proteomics of cells with *ELOVL5* knockdown had not intersected with other pathway sets.

Then, we analyzed alteration of the pathways that directly include *ELOVL5* and *IGFBP6* genes (Table 4). Specifically, *ELOVL5* was directly involved in the synthesis of very long-chain fatty acyl-CoAs and metabolism of linoleic and α-linolenic acids. According to the transcriptomic analysis, all these pathways were downregulated both upon *ELOVL5* and *IGFBP6* knockdowns; however, the majority of the differences were not statistically significant after multiple testing correction. The only exception was the “Reactome alpha linolenic acid ALA metabolism main pathway.” It was significantly downregulated upon *IGFBP6* knockdown. The majority of the same pathways were not regulated according to the proteomic analysis. However, the “Reactome linoleic acid LA metabolism main pathway” was downregulated upon *ELOVL5* knockdown and upregulated upon *IGFBP6* knockdown at the protein level, but these changes were insignificant after multiple testing correction. On the other hand, only two pathways included *IGFBP6* gene, and one of them (“Reactome regulation of IGF activity by IGFBP main pathway”) was activated in both knockdowns according to the proteomic analysis. However, after multiple testing correction, only the activation upon *IGFBP6* knockdown was significant. Overall, the pathway analysis indicates that the changes in the expression of one of the genes from the pair *ELOVL5*–*IGFBP6* can alter the pathways containing the other one; however, additional experiments are needed to prove this hypothesis.

**TABLE 4:** The pathways containing *ELOVL5* and *IGFBP6* genes which were regulated upon knockdown of these genes.

Pathway	KD *ELOVL5*			KD *IGFBP6*		
	Direction	*p*-value	FDR *p*-value	Direction	*p*-value	FDR *p*-value
**Transcriptomic analysis**						
Reactome linoleic acid LA metabolism main pathway	Down	0.002	0.111	Down	0.045	0.100
Reactome alpha linolenic acid ALA metabolism main pathway	Down	0.022	0.177	Down	0.007	0.032
Reactome synthesis of very long chain fatty acyl-CoAs main pathway	Down	0.002	0.107	Down	0.030	0.077
Reactome regulation of IGF activity by IGFBP main pathway	Up	< 0.001	0.074	Up	0.056	0.115
**Proteomic analysis**						
Reactome linoleic acid LA metabolism main pathway	Down	0.025	0.339	Up	0.014	0.054
Reactome alpha linolenic acid ALA metabolism main pathway	Down	0.193	0.614	Down	0.476	0.703
Reactome synthesis of very long chain fatty acyl-CoAs main pathway	Down	0.236	0.659	Up	0.525	0.759
Reactome regulation of IGF activity by IGFBP main pathway	Up	0.013	0.255	Up	0.007	0.035

### Activity of MMPs

**FIGURE 10:**
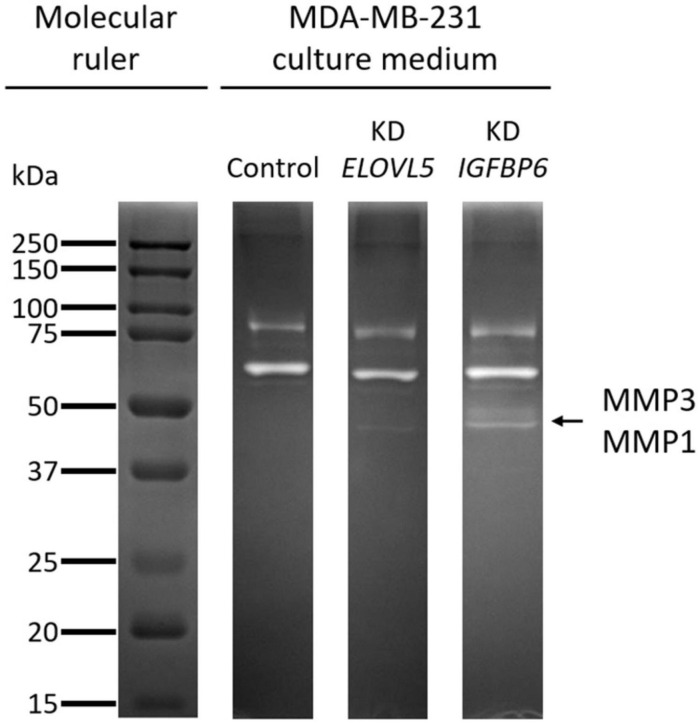
Zymogram of cell culture medium samples obtained after incubation with control MDA-MB-231 (LUC) cells and the cells with a stable knockdown of *ELOVL5* and *IGFBP6* genes.

The increase in the expression of MMPs MMP1 (molecular weight, 43 kDa) and MMP3 (molecular weight, 45 kDa), detected by transcriptomic and proteomic analysis, was further confirmed by zymography assessment of the activity of MMPs (Figure 10 and Supplementary Figure 10). It was shown that upon the knockdown of the *ELOVL5* gene, one pale band appears on the zymogram of the culture medium, which corresponds to the presence of active matrix metalloproteinase with a molecular weight of about 43 kDa (presumably MMP1). Upon the knockdown of the IGFBP6 gene, two bands appear on the zymogram of the culture medium, corresponding to the presence of active MMPs with molecular weights of about 43 and 45 kDa (presumably MMP1 and MMP3).

## Discussion

In this work, to study the changes in model tumor cells associated with the changes in the expression of the *ELOVL5* and *IGFBP6* genes, we decided to knock down the genes under consideration using RNA interference. We chose a stable knockdown with shRNA, as this method has been well developed to date and does not completely inhibit the expression of the selected gene. In contrast to various knockout methods, a stable shRNA-mediated knockdown partially decreases expression, which better reflects physiological changes observed *in vivo* (Boettcher and McManus, 2015). On the other hand, gene overexpression can often result in too high non-physiological levels of mRNA and protein of the selected gene, which also makes this method less suitable for this work (Prelich, 2012). Despite the fact that neither *ELOVL5* nor *IGFBP6* has substantial predictive power as single genes, we decided to perform separate knockdowns to reveal the impact of each of them on the behavior of BC cells and to find similarities and differences in their action. Therefore, it was necessary to choose a cell line with sufficiently high levels of expression of the studied genes.

According to a previously developed classification, such cells should form fewer metastases *in vivo* (Galatenko et al., 2015). This hypothesis is supported by previously published data on the ability of different BC cell lines to form lung metastases in immunodeficient mice *in vivo* (Valentiner et al., 2005). For example, MDA-MB-231 cells (green dot in Figure 1) formed metastases in lungs in only 14% of cases, while MCF7 cells (orange dot in Figure 1) with significantly lower *IGFBP6* gene expression formed metastases in 33% cases, and DU4475 cells (red dot in Figure 1), which, in addition, has a decreased expression of the *ELOVL5*, formed lung metastases in 86% cases (Valentiner et al., 2005).

Previously, we described generation of MDA-MB-231 cells with stable knockdown of *IGFBP6* gene (Nikulin et al., 2018). In this work, in addition, we generated MDA-MB-231 cells with stable knockdown of *ELOVL5* gene and analyzed different properties associated with metastatic potential.

Today, it is well known that the proliferation rate is an important indicator in assessing the metastatic potential of tumor cells. In particular, it was previously shown on model cell lines that an increase in the proliferation rate leads to an increase in the number of metastases *in vivo* (Hirabayashi et al., 1998; Marshall et al., 2004). These data are in agreement with clinical observations demonstrating that tumor doubling time, which depends on the rate of proliferation of tumor cells, affects relapse-free survival and overall survival (Tubiana, 1989). Our previous study revealed that the knockdown of *IGFBP6* increases the proliferation rate of MDA-MB-231 cells. This phenomenon can be explained by a classical IGF-dependent mechanism of action of the IGFBP6 protein. The knockdown of the *IGFBP6* gene leads to a decrease in the IGFBP6 protein content and to an increase in the content of free IGF-2 in the culture medium and stimulation of cell growth (Annunziata et al., 2011; Bach, 2016; Allard and Duan, 2018). In this work, we confirmed our previous finding and showed that, in contrast to the *IGFBP6* knockdown, the knockdown of *ELOVL5* has no effect on cell proliferation. An increase in the activity of cellular respiration and assembly of ribosomes found in the cells with the knockdown of the *IGFBP6* gene by means of proteomic analysis also indirectly indicates an increased proliferative activity. Moreover, we showed that the cells with *IGFBP6* mRNA knockdown are more resistant to apoptosis, probably also due to increased content of unbounded IGF-2.

Migration of tumor cells is an integral part of metastasis at almost every stage of the invasive-metastatic cascade, including local invasion. Previously, we demonstrated that knockdown of the *IGFBP6* gene leads to a sharp decrease in MDA-MB-231 cells’ migration in the transwell assay (Nikulin et al., 2018). In this work, we confirmed this finding with the help of a scratch assay and also demonstrated a similar effect for the *ELOVL5* gene. Observed changes in migratory activity were consistent with the conducted pathway and enrichment analysis, based on the transcriptomic and proteomic data.

Interestingly, the decrease in migratory activity as a result of the knockdown of the *IGFBP6* gene was more pronounced in the experiment with the transwell membrane inserts when compared to the scratch assay. It should be noted that the mechanisms of cell migration in these tests are fundamentally different from each other. Thus, in the scratch assay, gradients of chemoattractants are absent, and cells move collectively, interacting with each other and with extracellular matrix (ECM) proteins (Liang et al., 2007; Jonkman et al., 2014). This collective migration better reflects the *in vivo* situation. On the other hand, when considering membrane insert, the cell must completely lose contact with other cells and significantly change their shape during the passage of the pore, while the movement occurs along the gradient of chemoattractants, since FBS is present in the lower chamber (Chen, 2005).

The data indicate that the knockdown of the *IGFBP6* mRNA has a stronger impact on the ability of single cells to migrate through narrow spaces in comparison to collective migration. These results are in good agreement with previously published data, indicating that invasion is associated with the arrest of the cell cycle, and therefore, the migration activity of rapidly proliferating cells should be lower (Kohrman and Matus, 2017). At the same time, previous modeling showed that the metastatic potential of cells with a high proliferation rate is often higher than that of the cells with an increased ability to invade (Hecht et al., 2015).

From the classical point of view, malignant cells in the course of spreading through the body lose their contact with neighboring cells and become more mobile (Janiszewska et al., 2020). Thus, the loss of adhesion should be associated with a more aggressive phenotype. It is well known that a lot of adhesion molecules play an important role in the progression of cancer (Lange et al., 2014; Samatov et al., 2016). However, it is worth noting that cells often migrate collectively, and, in this case, they are not characterized by a complete loss of intercellular contacts (Janiszewska et al., 2020). The most striking example is the process of epithelial-to-mesenchymal transition (EMT), manifested by loss of E-cadherin and the acquisition of mobility by tumor cells (Nieto et al., 2016). The formation of spheroids by many BC cell lines is also known to depend on the expression of E-cadherin (Iglesias et al., 2013). At the same time, the level of E-cadherin expression in tumor tissue is significantly associated with the prognosis of many types of cancer, including BC (low level is associated with a poor prognosis) (Rossetti et al., 2015).

MDA-MB-231 cells express E-cadherin at a rather low level and form loose spheroids (Ivascu and Kubbies, 2007). It is known that the aggregation of cells into spheroids can also depend on other adhesion molecules, such as CDH3 (Stadler et al., 2018) and CD44 (Suarez et al., 2019). What is more, the expression of many adhesion molecules that can potentially participate in the formation of intercellular contacts in spheroids is associated with the prognosis of the disease. In particular, low expression of claudin (one of the structural components of tight junctions) in BC cells is associated with a poor prognosis (Rossetti et al., 2015). Thus, the study of intercellular adhesion can be useful in assessing the metastatic potential of cells.

The data obtained in this work indicate that, as a result of the knockdown of the *ELOVL5* and *IGFBP6* mRNAs in MDA-MB-231 cells, the expression of a number of adhesion molecules (such as *CDH11*, *CLDN1*, and *DSP*) decreases, which in turn leads to the disruption of cell–cell contacts. Furthermore, the relationship between the expression levels of the *CDH11* gene, the *ELOVL5* and *IGFBP6* genes, is the same in clinical BC samples as in our *in vitro* model. It is known that CDH11 is one of the classic type 2 cadherins, which plays an important role in the formation of intercellular contacts during osteogenesis (Piao et al., 2017). Interestingly, the increased expression of CDH11 can stimulate the invasion of some types of tumor cells (e.g., prostate cancer cells) and reduce the proliferation rate and ability to invade for other types of tumors (e.g., head and neck tumors). In this work, we ascertained that a decreased expression of the *CDH11* gene in BC cells may be associated with a more aggressive phenotype.

Matrix metalloproteinases are zinc-dependent extracellular endopeptidases involved in the remodeling of the ECM, both in normal conditions and in various pathologies, including malignant neoplasms (Gialeli et al., 2011; Cathcart et al., 2015). MMPs have different substrate specificities. In particular, MMP1 belongs to the family of collagenases, which predominantly break down various types of collagens and gelatin (denatured collagen), and MMP3 belongs to the family of stromelysins, which break down proteoglycans, laminins, fibronectin, and some types of collagens (Overall, 2002). It is also remarkable that several MMPs can regulate the availability of various growth factors to cells. For example, MMP1 can degrade the proteins IGFBP3 and IGFBP5, which bind IGFs, thereby increasing the concentration of the latter. MMP3 can also cleave IGFBP3, resulting in a similar effect.

To date, it is known that the increased expression of the MMP1 protein in tumor tissue is associated with metastatic lesions of lymph nodes in BC, and a decrease in MMP1 gene expression reduces the metastatic potential of BC cells both *in vitro* and *in vivo* in animal experiments (Liu et al., 2012; Wang et al., 2018). High MMP3 expression is also associated with a poor prognosis for BC (Mehner et al., 2015). Thus, the increase in MMP1 and MMP3 expression observed after the knockdown of the *ELOVL5* and *IGFBP6* genes is consistent with the hypothesis that ELOVL5 and IGFBP6 are associated with tumor metastatic potential. Moreover, the expression of the *MMP1* mRNA and the *ELOVL5–IGFBP6* pair of mRNAs is interrelated in patient tumors, and the direction of the change in expression is the same with our *in vitro* model. However, the conducted correlation analysis showed that the regulation of *MMP3* gene expression *in vivo* in patients’ tumors may differ significantly from the pattern we observed *in vitro*.

Overall, the knockdown of *ELOVL5* had a number of seemingly unexpected consequences. For example, the decreased expression of the enzyme involved in fatty acids (FAs) elongation influenced cell migration, cell–cell interactions, and MMP’s synthesis. However, this is not an entirely unexpected result, as previously, it was shown that omega-3 and omega-6 PUFAs, the products of ELOVL5 activity, affect the proliferation, migration, and invasion of cancer cells *in vitro* (Chamras et al., 2002; Yun et al., 2014; Gonzalez-Reyes et al., 2017; Huang et al., 2017) and that dietary omega-3 FAs reduce the risk of BC development, as well as the risk of its relapse (Abdelmagid et al., 2016; Playdon et al., 2017; Romieu et al., 2017; Shapira, 2017). Still, there is no simple explanation for these results, since the effect of PUFAs on cellular processes is multifaceted.

First of all, PUFAs are incorporated into membrane phospholipids and influence their fluidity and selective permeability and functioning of membrane receptors (Wiktorowska-Owczarek et al., 2015). PUFAs also can modulate the activity of different transcriptional factors (Jump et al., 1996). Furthermore, docosahexaenoic acid (DHA, omega-3 PUFA) and arachidonic acid (AA, omega-6 PUFA) have a wide range of bioactive metabolites acting as local hormones or signaling molecules and regulate cell proliferation, adhesion, migration, angiogenesis, vascular permeability, and inflammatory responses [role of DHA metabolites resolvins, protectins, and maresins is reviewed in Kuda (2017) and the role of AA metabolites eicosanoids, prostaglandins, and leukotrienes is reviewed in Tallima and El Ridi (2018)]. There is also evidence of the direct inhibition of MMPs activity by PUFAs (Nicolai et al., 2017), although information on this issue is controversial (Liuzzi et al., 2007). The inhibition effect of omega-6 PUFAs on *MMPs* expression was shown *in vivo* in a coronary heart disease-induced rat model (Lu et al., 2018), but the mechanism is unclear.

The association between IGFBP6 and cancer appears to be more obvious, as a large number of studies on the role of the IGF/IGF1R signaling pathway in oncogenesis have been carried out to date (Trajkovic-Arsic et al., 2013; Brouwer-Visser and Huang, 2015; Salisbury and Tomblin, 2015; Vigneri et al., 2015; Tracz et al., 2016). At the same time, significant differences in the primary structure of seven IGFBPs, in their posttranslational modifications and in their tissue specificity, indicate differences in their functions. Differences in IGFBPs structures also indicate that their action is not limited to the inhibition of IGFs. This is confirmed by the fact that, for some IGFBPs, the IGF- and IGF1R-independent action on cells has been demonstrated (Firth and Baxter, 2002).

For many IGFBPs, their role in various pathological processes was demonstrated, including cancer [the *IGFBP6* gene is differentially expressed in nasopharyngeal carcinoma (Chen et al., 2016); the plasma protein level of IGFBP6 changes with ovarian cancer (Gunawardana et al., 2009; Wang et al., 2013); *IGFBP6* mRNA and protein levels are significantly lower in colorectal cancer (CRC) tissues and low *IGFBP6* expression correlated with poor overall survival (Zhao et al., 2020)]. Knockdown of *IGFBP6* in HT-29, Caco-2, SW620, and HCT116 cells influenced proliferation, migration, and invasion (Zhao et al., 2020).

It was shown that IGFBP6 acts on different cancer cell lines both by the inhibition of IGFs and by IGF-independent mechanisms in an autocrine and/or paracrine fashion. Information about IGFBP6 and its effects on cellular processes is reviewed in Bach (2016). Interestingly, that IGFBP6 contains a nuclear localization signal, which targets it to the nucleus, where it regulates gene expression (Poreba and Durzynska, 2020). IGFBP6 showed its ability to bind the *EGR1* promoter and induce its activity in stably transfected nasopharyngeal cancer (NPC) cell lines overexpressing IGFBP6 (Kuo et al., 2010). The increased expression of IGFPB6 inhibited the proliferation, invasion, and metastatic activity of the NPC cells, suggesting that IGFBP6 acts as a tumor suppressor (Kuo et al., 2010). In our study, expression of *TOE1* (target of EGR1, member 1) after *IGFBP6* knockdown increased 2.7 times (FDR *p* = 6.2 × 10^–6^), suggesting this mechanism can play an important role in BC, too.

## Conclusion

In conclusion, it can be assumed that low expression of the *ELOVL5* and *IGFBP6* genes leads to the stimulation of BC cell invasion at the first stage of the invasive metastatic cascade due to the increased proliferation rate, more efficient decomposition of the ECM by MMPs, and the weakening of cellular junctions. Increased resistance to apoptosis may also play an important role in the spreading of tumor cells throughout the body. Further research will help shed light on the detailed molecular mechanisms responsible for the observed changes in tumor cell properties resulting from a decreased expression of the *ELOVL5* and *IGFBP6* genes.

## Data Availability Statement

The datasets presented in this study can be found in online repositories. The names of the repository/repositories and accession number(s) can be found below: https://www.ebi.ac.uk/pride/archive/, PXD023892 and https://www.ncbi.nlm.nih.gov/geo/, GSE165854.

## Author Contributions

AT supervised the study. SNi, GZ, AP, US, and AT designed the study. SNi, GZ, MR, DW, SNe, JB, GB, and LA performed the experiments and analyzed the data. SNi and GZ wrote the manuscript. AT and AP revised the manuscript. All authors contributed to the article and approved the submitted version.

## Conflict of Interest

The authors declare that the research was conducted in the absence of any commercial or financial relationships that could be construed as a potential conflict of interest.
