# Supplementary material for: Effect of the Expression of ELOVL5 and IGFBP6 Genes on the Metastatic Potential of Breast Cancer Cells
Source: Front Genet. 2021 Jun 2;12:662843. doi: 10.3389/fgene.2021.662843 (PMC8206645; doi:10.3389/fgene.2021.662843)
Supplement: Supplementary file 1 [file Data_Sheet_1.PDF]

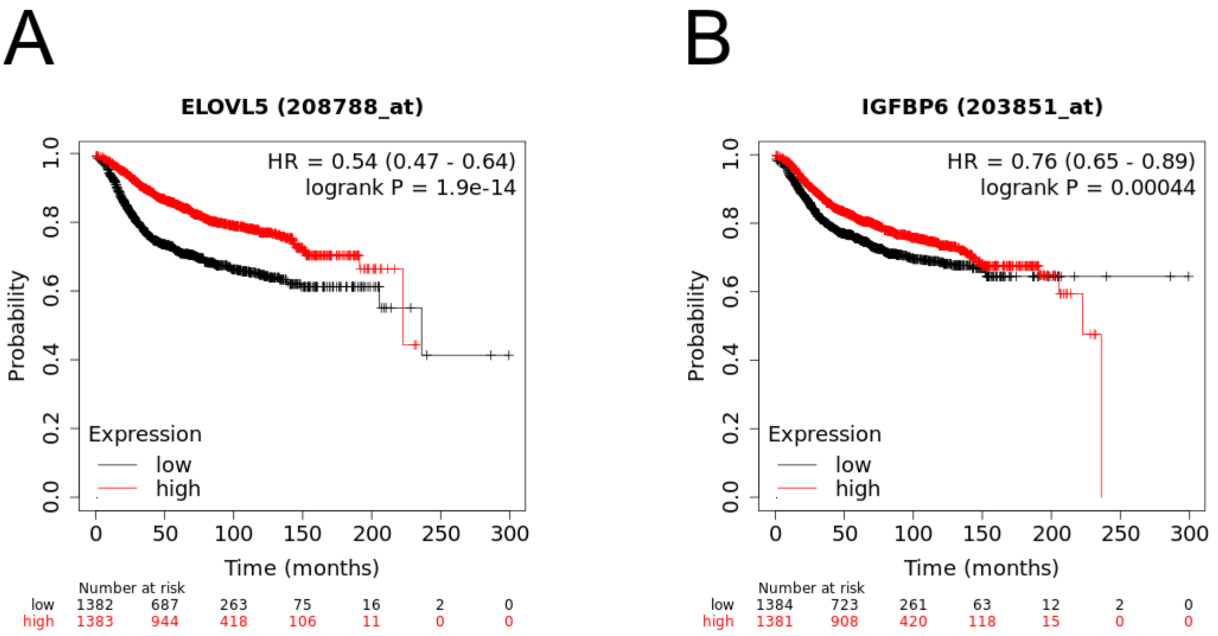

Figure S1. Kaplan-Meier plots of distant metastasis free survival (DMFS) of breast cancer patients with a different expression of *ELOVL5* **(A)** and *IGFBP6* **(B)** genes (according to kmplot.com).

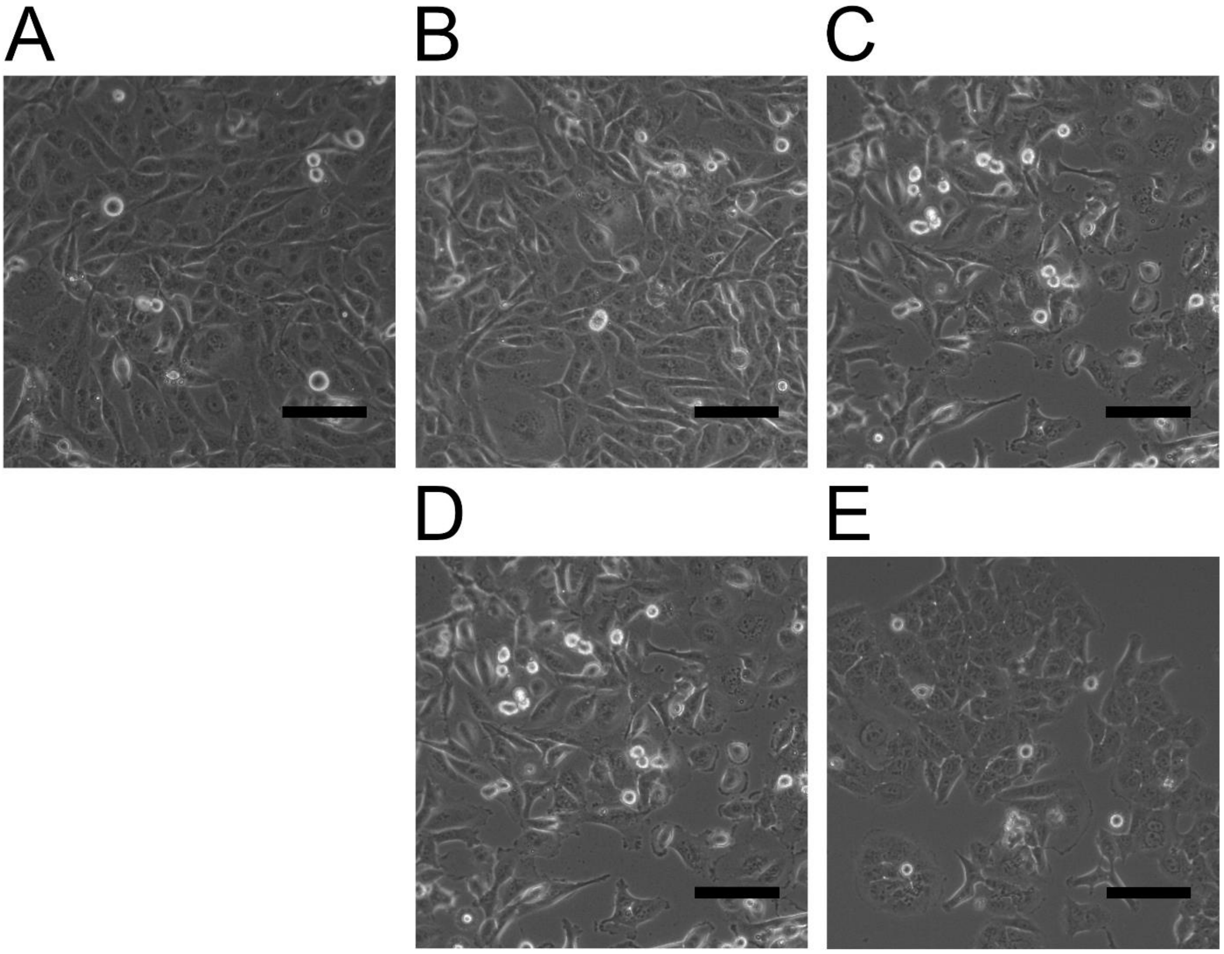

Figure S2. Photo of MDA-MB-231 (LUC) **(A)**, MDA-MB-231 (ELOVL5_1) **(B)**, MDA-MB-231 (ELOVL5_2) **(C),** MDA-MB-231 (IGFBP6_1) **(D)** MDA-MB-231 (IGFBP6_2) **(E)** cells. The scale bar length is 100 µm.

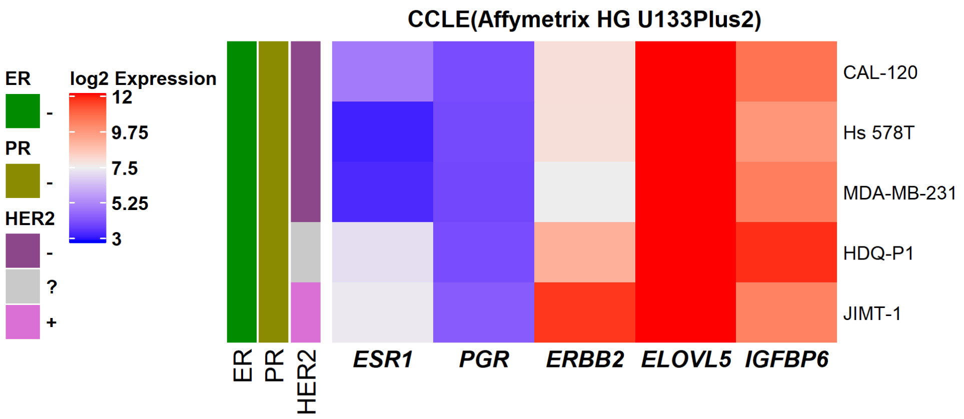

Figure S3. Expression of major molecular markers in breast cancer cell lines suitable for knockdown of *ELOVL5* and *IGFBP6* genes according to CCLE and (Dai et al., 2017). ER – estrogen receptor (gene *ESR1*), PR – progesterone receptor (gene *PGR*), HER2 - human epidermal growth factor receptor 2 (gene *ERBB2*).

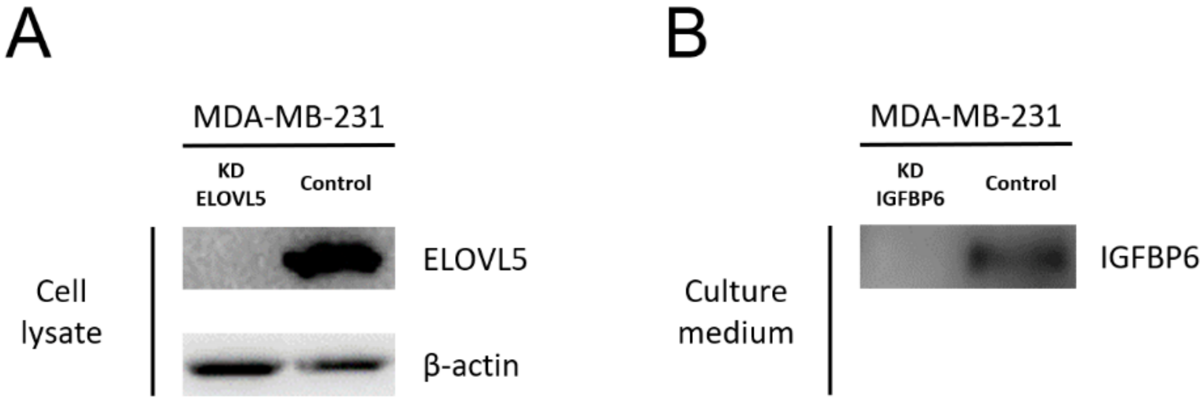

Figure S4. Additional results (the second experiment repetition) of analysis of the expression of ELOVL5 and IGFBP6 proteins by western blotting. **(A)** Relative expression of the ELOVL5 protein in the MDA-MB-231(ELOVL5) cell line compared to the control cell line. **(B)** Relative content of the IGFBP6 protein in the conditioned cell culture medium of MDA-MB-231(IGFBP6) cell line compared to the control cell line (samples were normalized to the number of cells).

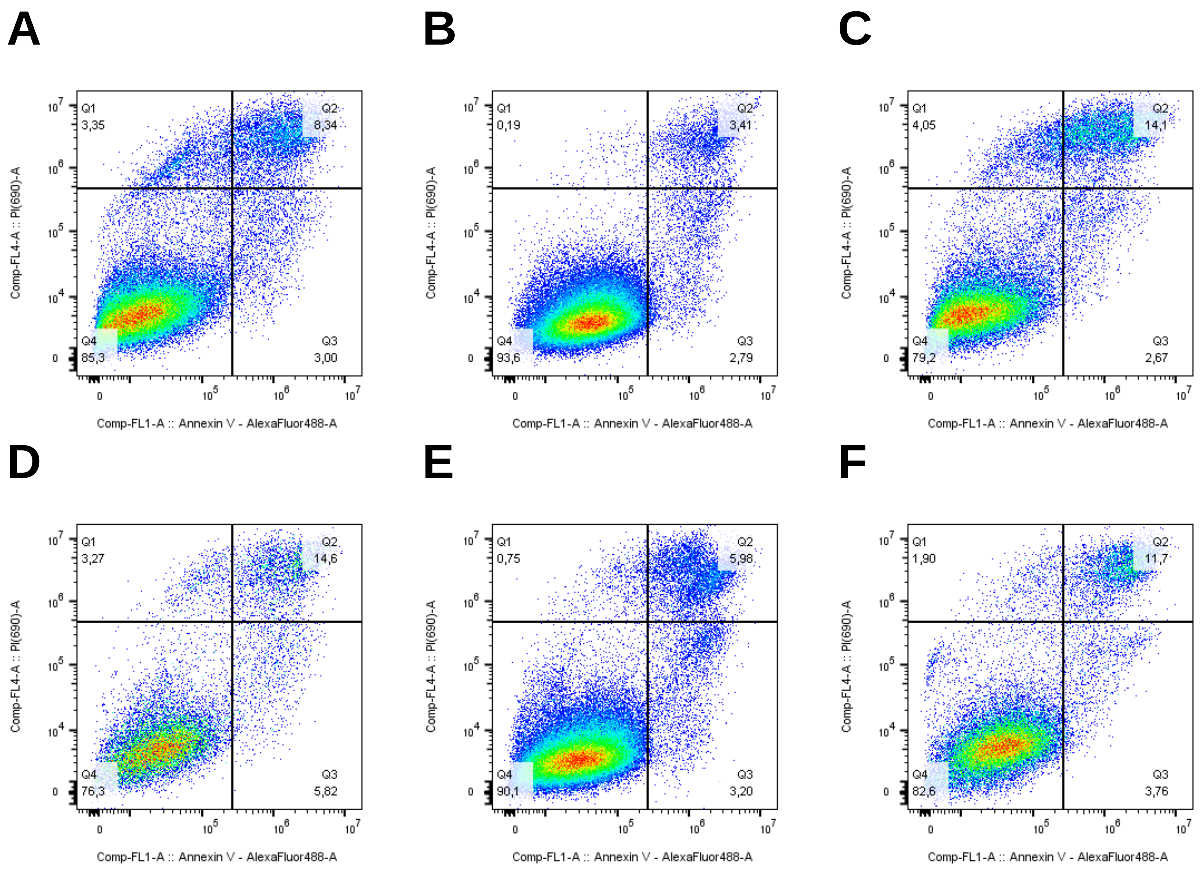

Figure S5. Effect of *ELOVL5* and *IGFBP6* knockdown on the activation of apoptosis (the second and the third experiment repetitions). Two-dimensional plots of the integral fluorescence intensity of the annexin V conjugate with Alexa Fluor 488 dye (horizontal axis) and the integral fluorescence intensity of propidium iodide (vertical axis) in MDA-MB-231 cells with *ELOVL5* **(A, D)** and *IGFBP6* **(B, E)** genes knockdown, as well as in controls cells **(C, F)**.

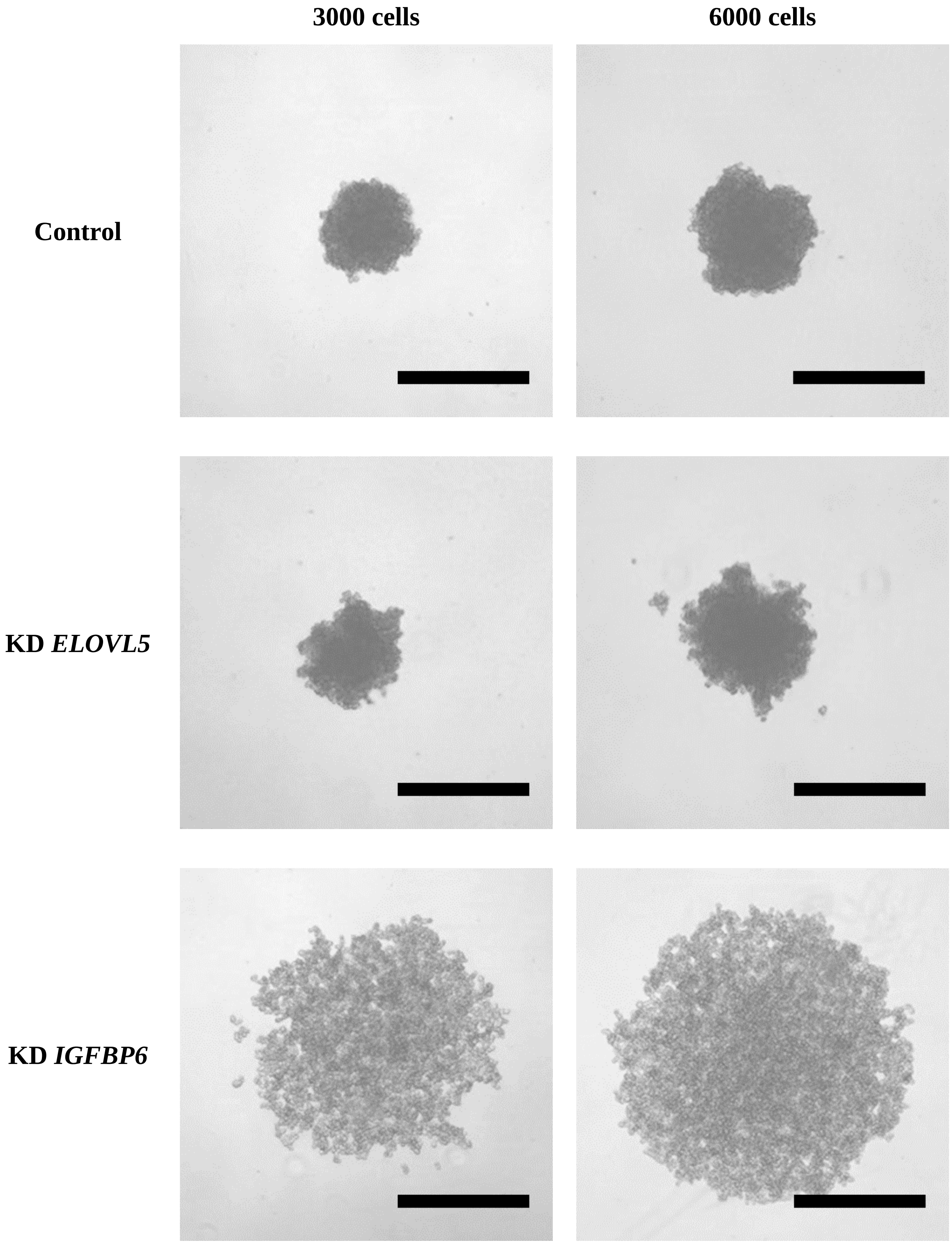

Figure S6. Photo of 3D cell spheroids (3000 and 6000 cells per well at zero time point) after 96 h from seeding consisting of control cells MDA-MB-231(LUC) and the cells with knockdown of *ELOVL5* and *IGFBP6* genes. The scale bar length is 200 µm.

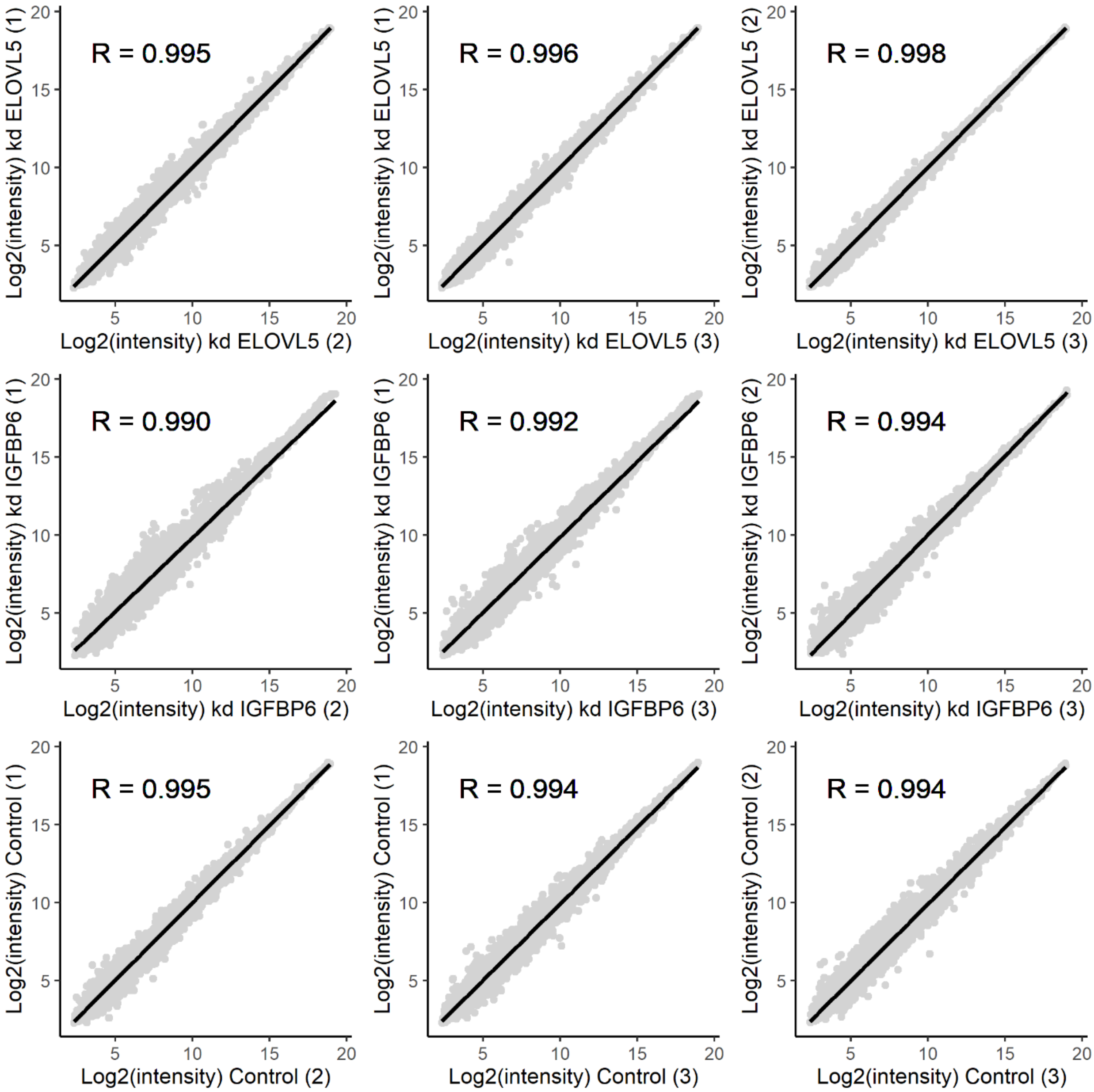

Figure S7. Correlation between different replicates in transcriptomics analysis (Affymetrix).

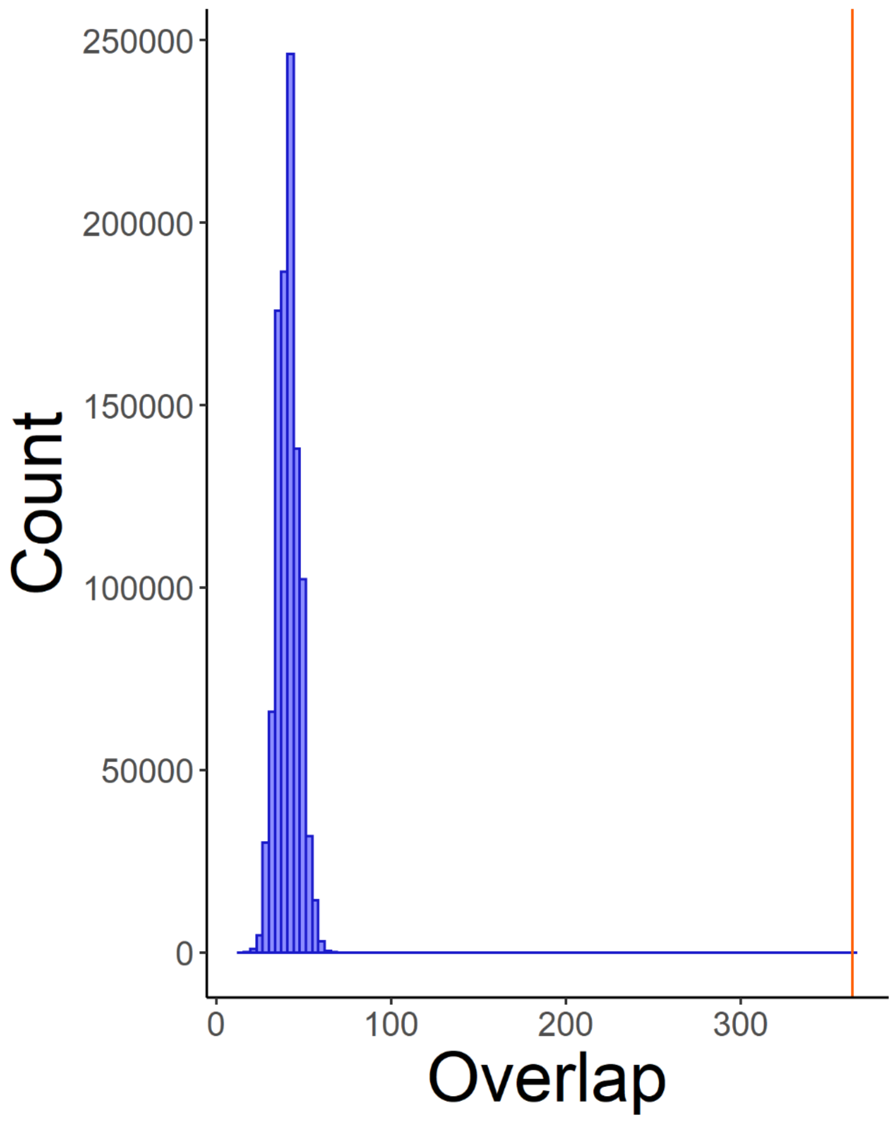

Figure S8. The results of the permutation test (n = 1 000 000). The distribution of the number of the genes which significantly change their expression in the same direction after the knockdown of *ELOVL5* and *IGFBP6* genes in case of completely independent changes is shown in purple. Actual size (364 genes) of the observed in the experiment overlap between significantly regulated in the same direction genes is shown as orange line. Maximal size of the random overlap after 1 000 000 permutations was 74.

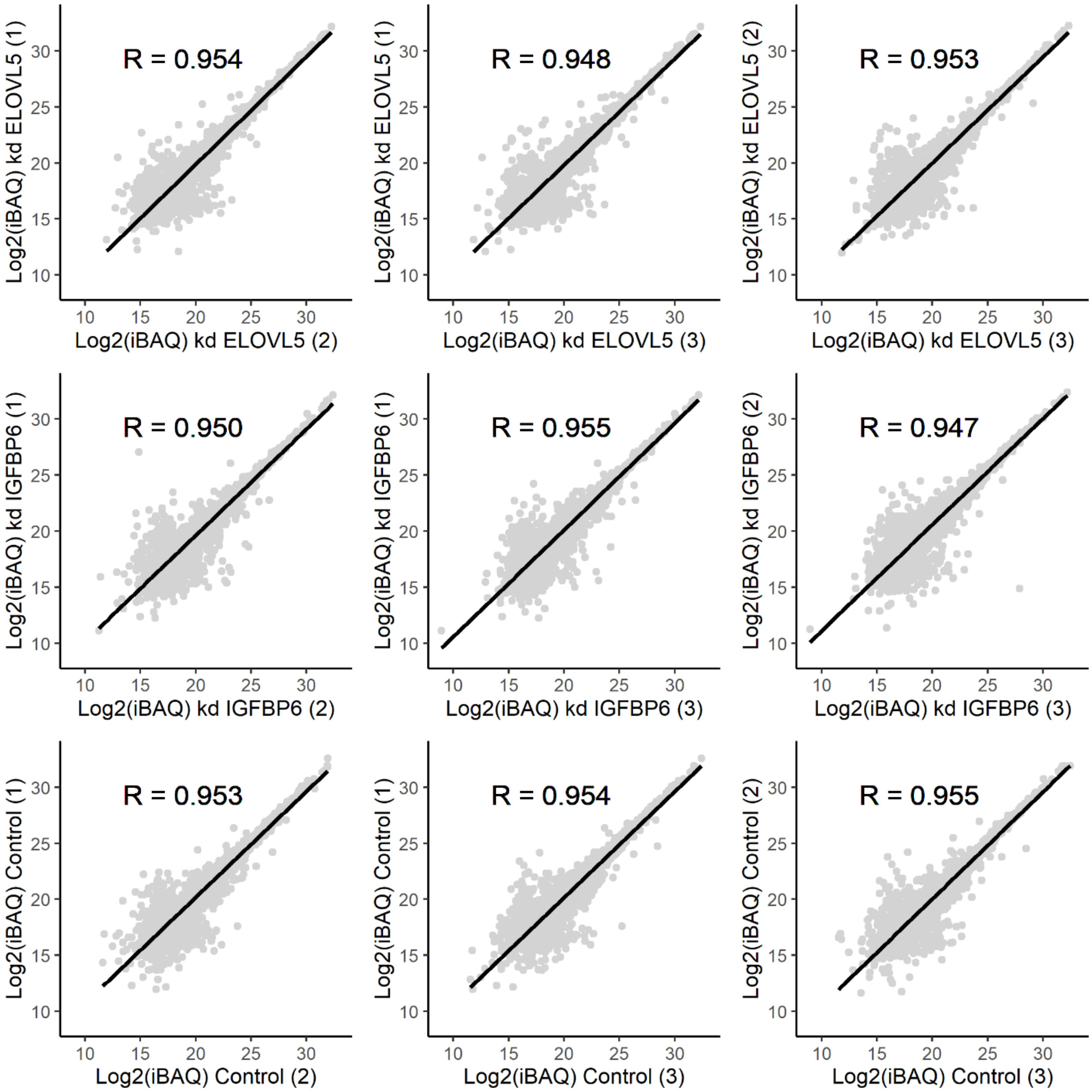

Figure S9. Correlation between different replicates in proteomic analysis (iBAQ).

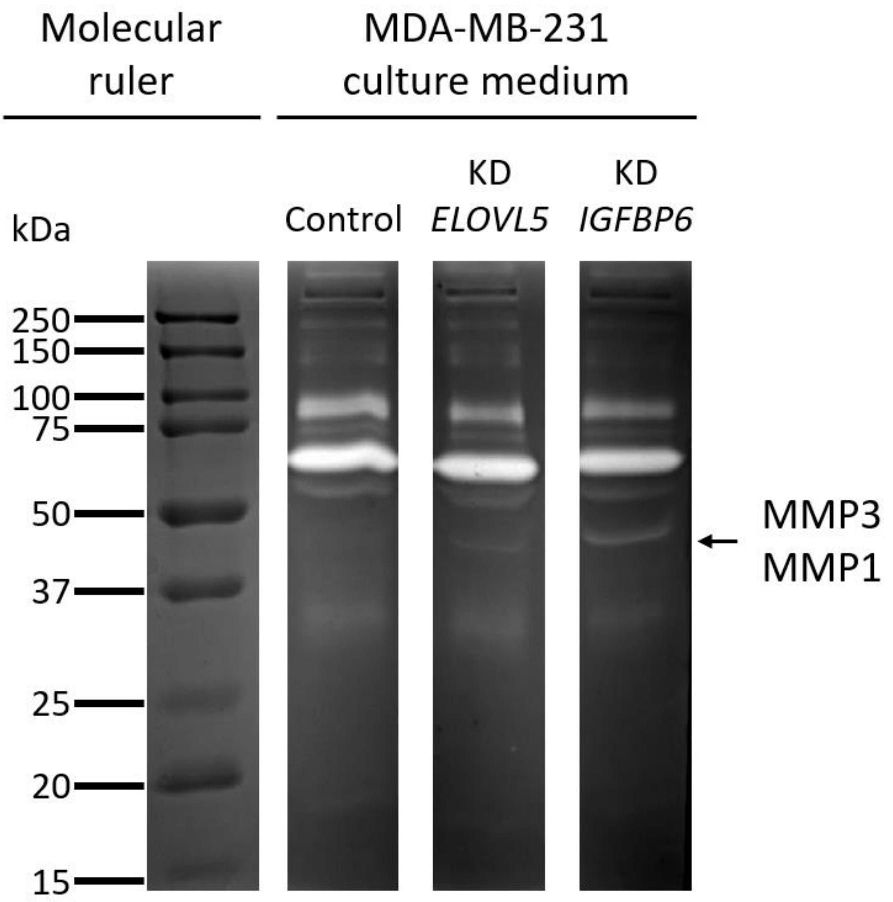

Figure S10. Zymogram (the second experiment repetition) of cell culture medium samples obtained after incubation with control MDA-MB-231(LUC) cells and the cells with knockdown of *ELOVL5* and *IGFBP6* genes.

Table S1. Properties of the utilized breast cancer transcriptomic data sets.

Dataset	Platform	Number of samples (Estrogen Receptor status)	Dataset ID
cBioMETABRIC	Illumina HumanHT-12 v3	445(-)	1
		1459(+)	2
GSE102484	Affymetrix HG U133Plus2	283(-)	3
		400(+)	4
GSE12093	Affymetrix HG U133A	136(+)	5
GSE17705	Affymetrix HG U133A	298(+)	6
GSE22220	Illumina humanRef-8 v1_0	82(-)	7
		134(+)	8
GSE3494	Affymetrix HG U133A Affymetrix HG U133B	34(-)	9
		213(+)	10
		4(?)	N/A
GSE58644	Affymetrix HG 1_0 ST	70(-)	11
		250(+)	12
		1(?)	N/A
GSE6532	Affymetrix HG U133Plus2	87(+)	13
	Affymetrix HG U133A Affymetrix HG U133B	45(-)	14
		262(+)	15
		20(?)	N/A
TCGA BRCA	Illumina HiSeq 2000	261(-)	16
		897(+)	17
		63(?)	N/A

Table S2. Sequences for shRNA.

Target sequence	Oligonucleotides for shRNA	Generated cell line
ELOVL5:<br>GCGGAAGGATTGAAGTCAA	**Upper chain:**<br>5'-gatccGCGGAAGGATTGAAGTCAATTCAAGAGATTGACTTCAATCCTTCCGCTTTTTTACGCGTg-3'<br>**Lower chain:**<br>5'-aattcACGCGTAAAAAAGCGGAAGGATTGAAGTCAATCTCTTGAATTGACTTCAATCCTTCCGCg-3'	MDA-MB-231 (ELOVL5_1)
ELOVL5:<br>GCTGAACATCTGGTGGTTT	**Upper chain:**<br>5'-gatccGCTGAACATCTGGTGGTTTTTCAAGAGAAAACCACCAGATGTTCAGCTTTTTTACGCGTg-3'<br>**Lower chain:**<br>5-aattcACGCGTAAAAAAGCTGAACATCTGGTGGTTTTCTCTTGAAAAACCACCAGATGTTCAGCg-3'	MDA-MB-231 (ELOVL5_2)

Table S3. Oligonucleotide primers for RT-PCR.

Gene	Sequences	Length of the amplicon	Amplification efficiency (±SEM)
ELOVL5	**Forward:**<br>5'-CATTCAGACCTACAACAAGAAAGGG-3'<br>**Reverse:**<br>5'-AGGGGTGAAAAGCTGTTGGT-3'	111 bp	2.03±0.01
MMP1	**Forward:**<br>5'-TTTGCCGACAGAGATGAAGTCCG-3'<br>**Reverse:**<br>5'-AGGGAAGCCAAAGGAGCTGTAGA-3'	114 bp	1.94±0.12
MMP3	**Forward:**<br>5'-ATCTCTTCCTTCAGGCGTGGATGC-3'<br>**Reverse:**<br>5'-CCAGCTCGTACCTCATTTCCTCTGA-3'	111 bp	1.94±0.02
EEF1A1	**Forward:**<br>5'-CCCTAAAAGCCAAAATGGGAAA-3'<br>**Reverse:**<br>5'-TAGTGGTGGACTTGCCCGAAT-3'	84 bp	1.98±0.15
HUWE1	**Forward:**<br>5'-GCCTGACCTGAGTGGGTTAGTG-3'<br>**Reverse:**<br>5'-CACACTGCTCCAACAGCTTCC-3'	84 bp	1.97±0.07

Table S4. Statistics on the number of differentially expressed genes after knockdown of the *ELOVL5* and *IGFBP6* genes in comparison with control cells.

**Knockdown of *ELOVL5***		
	Absolute number	Proportion, %
Increased expression	274	1,02
Decreased expression	212	0,79
**Knockdown of *IGFBP6***		
	Absolute number	Proportion, %
Increased expression	2319	8,65
Decreased expression	2217	8,27

Table S5. The results of the correlation analysis. Statistically significant correlations are marked in bold.

**Dataset ID**	**Correlation coefficients and FDR p-values**											
	ELOVL5						***IGFBP6***					
	MMP1		***MMP3***		***CDH11***		***MMP1***		***MMP3***		***CDH11***	
	R	***p***	***R***	***p***	***R***	***p***	***R***	***p***	***R***	***p***	***R***	***p***
1	0.081	1.56E-01	0.044	4.74E-01	**0.415**	**2.71E-18**	0.023	7.70E-01	**0.224**	**1.13E-05**	**0.583**	**1.28E-39**
2	-0.040	1.69E-01	-0.026	3.80E-01	**0.222**	**6.85E-17**	**-0.107**	**1.24E-04**	**0.335**	**2.58E-38**	**0.341**	**1.18E-39**
3	**-0.158**	**2.41E-02**	0.140	5.07E-02	**0.262**	**6.61E-05**	**-0.373**	**6.72E-10**	0.110	9.31E-02	**0.420**	**1.69E-12**
4	**-0.191**	**1.15E-03**	-0.031	7.06E-01	0.027	6.93E-01	**-0.325**	**1.78E-10**	0.079	1.48E-01	-0.020	3.08E-01
5	0.036	7.30E-01	0.156	1.03E-01	**0.358**	**6.86E-05**	-0.003	9.82E-01	**0.354**	**1.68E-04**	**0.376**	**5.88E-05**
6	**-0.158**	**2.24E-02**	-0.070	3.26E-01	-0.050	5.07E-01	-0.127	6.33E-02	**0.224**	**6.62E-04**	**0.284**	**1.00E-05**
7	-0.221	1.56E-01	0.163	3.31E-01	0.144	4.05E-01	**-0.453**	**4.36E-04**	0.073	7.37E-01	0.137	4.72E-01
8	**-0.276**	**5.98E-03**	-0.081	5.12E-01	0.100	4.00E-01	-0.172	2.23E-01	0.157	2.82E-01	0.089	6.09E-01
9	0.192	6.33E-01	0.493	8.62E-02	**0.553**	**4.61E-02**	0.059	9.10E-01	0.253	4.59E-01	**0.565**	**1.92E-02**
10	**-0.385**	**2.64E-07**	-0.110	1.87E-01	0.071	4.27E-01	**-0.170**	**4.24E-02**	**0.410**	**1.50E-08**	**0.287**	**1.85E-04**
11	-0.023	9.21E-01	0.248	1.40E-01	0.231	1.73E-01	-0.032	8.94E-01	0.068	7.64E-01	0.232	1.89E-01
12	**-0.143**	**3.83E-02**	0.001	9.94E-01	**0.296**	**8.12E-06**	-0.032	6.89E-01	0.123	8.41E-02	**0.147**	**3.65E-02**
13	-0.191	3.49E-01	-0.053	8.39E-01	0.071	7.77E-01	0.086	6.74E-01	**0.299**	**3.98E-02**	**0.444**	**6.02E-04**
14	0.041	9.06E-01	0.272	2.98E-01	0.226	4.29E-01	-0.330	1.25E-01	-0.039	8.94E-01	0.192	4.72E-01
15	**-0.225**	**1.38E-03**	-0.113	1.26E-01	**0.151**	**3.65E-02**	-0.091	2.84E-01	**0.322**	**3.11E-06**	**0.337**	**7.94E-07**
16	-0.007	9.42E-01	0.093	2.34E-01	**0.291**	**1.09E-05**	**-0.264**	**8.00E-05**	-0.056	4.74E-01	0.104	1.73E-01
17	**-0.113**	**1.88E-03**	**-0.087**	**2.11E-02**	0.044	2.92E-01	**-0.268**	**1.66E-15**	0.037	3.29E-01	**-0.092**	**9.57E-03**

Table S6. Statistics on the number of differentially expressed proteins after knockdown of the *ELOVL5* and *IGFBP6* genes in comparison with control cells.

**Knockdown of *ELOVL5***		
	Absolute number	Proportion, %
Increased expression	5	0,12
Decreased expression	2	0,05
**Knockdown of *IGFBP6***		
	Absolute number	Proportion, %
Increased expression	454	10,96
Decreased expression	474	11,44
